# Supplementary material for: TRIM16 controls assembly and degradation of protein aggregates by modulating the p62‐NRF2 axis and autophagy
Source: EMBO J. 2018 Aug 24;37(18):e98358. doi: 10.15252/embj.201798358 (PMC6138442; doi:10.15252/embj.201798358)
Supplement: Supplementary file 1 — Appendix [file EMBJ-37-e98358-s001.pdf]

# Appendix

## TABLE OF CONTENTS

1. Appendix Figure S1
2. Appendix Figure S2
3. Appendix Figure S3
4. Appendix Figure S3 Legends
5. Appendix Figure S4

## Appendix Supplementary Figure 1

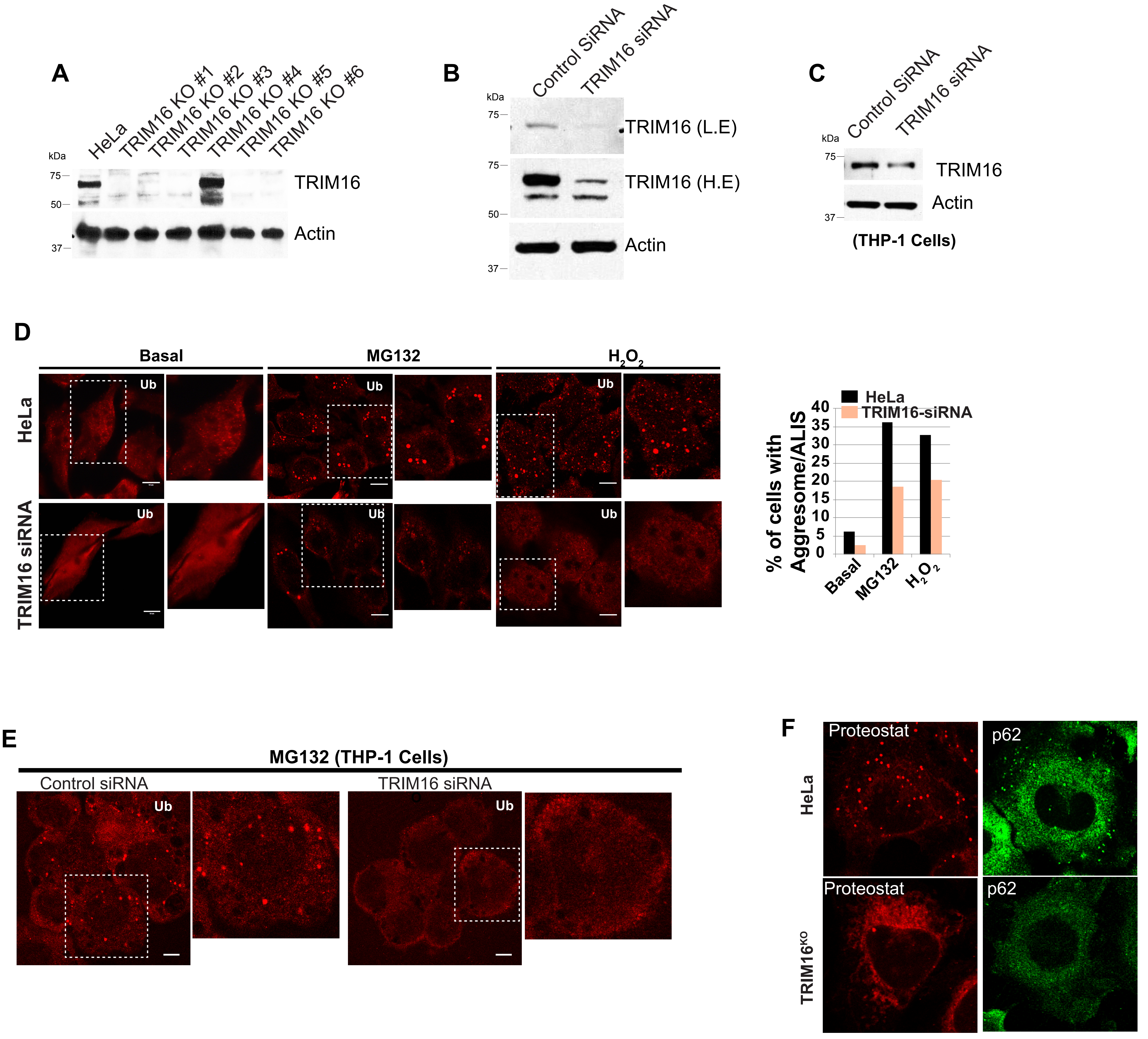


**Appendix Supplementary Figure 1.** (**A**) Western blot analysis of Hela and TRIM16 CRISPR-CAS9 knock out clones. (**B, C**) Western blot analysis of control and TRIM16 siRNA transfected (**B**) HeLa cells (**C**) THP-1. L.E, Low exposure; H.E, High exposure. (**D**) Left panel, Representative confocal images of control and TRIM16 siRNA transfected cells, untreated or treated with MG132 (20 µM, 2h), $H_2O_2$ (200 µM, 2h), and processed for immunofluorescence microscopy (IF) analysis with Ubiquitin (FK2) antibody. Right panel, HeLa and $TRIM16^{KO}$ cells were visualized and cells with more than 2 visible aggresomes/ALIS were considered positive. (**E**) Representative confocal images of control and TRIM16 siRNA transfected THP-1 cells treated with MG132 (20 µM, 2h) and the samples were processed for IF analysis with FK2. (**F**) Representative confocal images HeLa and $TRIM16^{KO}$ cells treated with $H_2O_2$ (200 µM, 2h) and processed for IF analysis with Proteostat dye and p62 antibody.

# Appendix Supplementary Figure 2

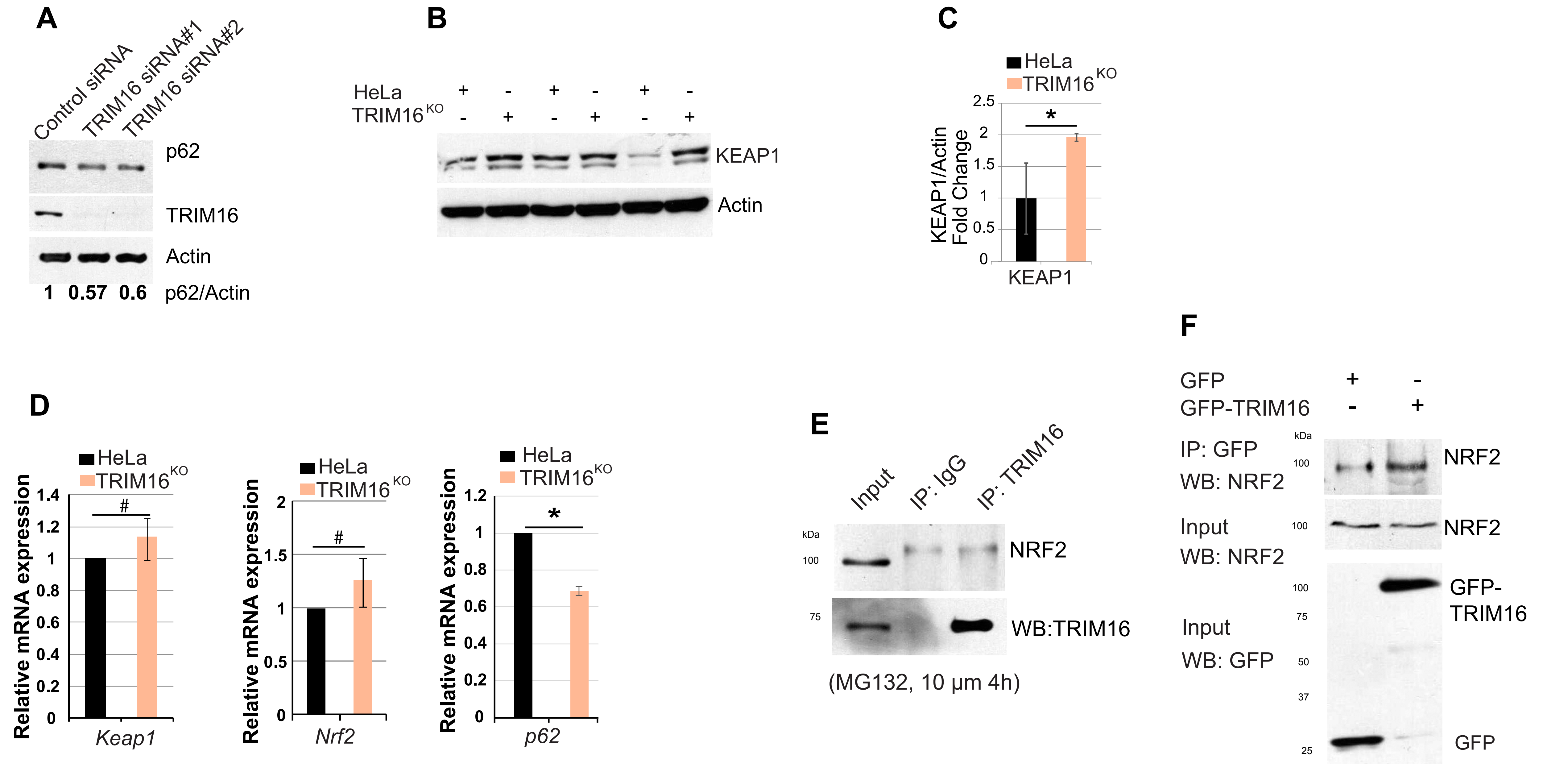


**Appendix Supplementary Figure 2.** (**A**) Protein extracted from control and TRIM16 siRNA transfected cells are subjected to western blotting with indicated antibodies. (**B**) Western blot analysis of HeLa and $TRIM16^{KO}$ cell lysates probed with indicated antibodies. (**C**) Densitometric analysis (mean ±SD) of protein band intensity relative to Actin, n=3, *p=0.05, (Student's unpaired t test). (**D**) RNA isolated from HeLa and $TRIM16^{KO}$ cells were subjected to quantitative to real-time PCR (qRT-PCR) with primers of p62, Keap1, and Nrf2. Data represent mean ±SD, n=3, *p < 0.05; #insignificant. (**E**) IP analysis of interaction between endogenous TRIM16 and endogenous NRF2 in HeLa cell lysates in presence of MG132. (**F**) Co-IP analysis of interaction between TRIM16 and NRF2 in HEK-293T lysates of cells expressing GFP or GFP-TRIM16.

# Appendix Supplementary Figure 3

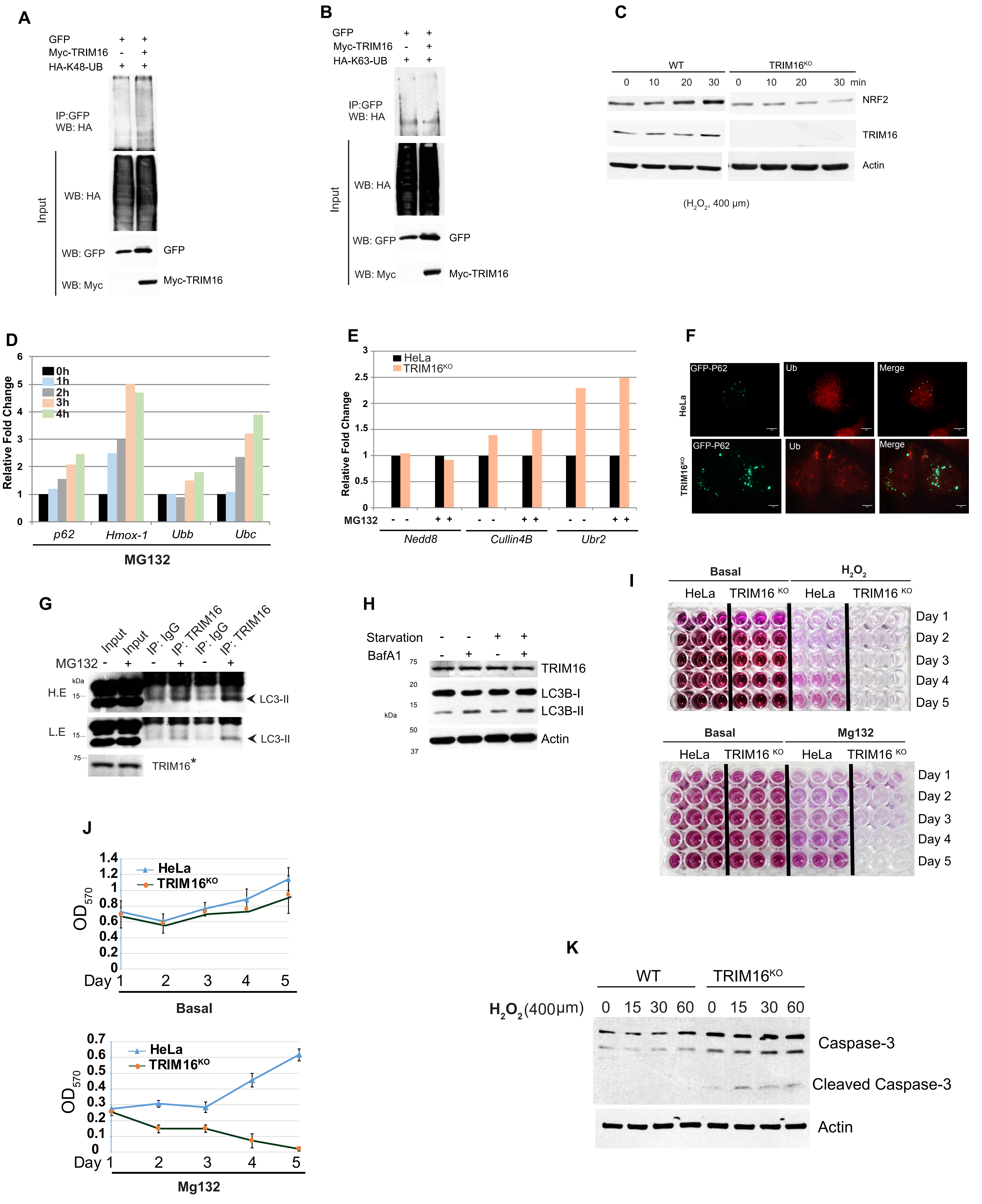

# Appendix Supplementary Figure 3

**Appendix Supplementary Figure 3**. (**A, B**) Analysis of GFP ubiquitination in absence and presence of TRIM16 by Co-IP assays using transiently transfected plasmid constructs as indicated. Two different variants of Ubiquitin protein are used, one that can only be ubiquitinated at Lysine 48 residue (HA-K48-UB) and other that can be only ubiquitinated at Lysine 63 residue (HA-K63-UB). All other lysine residues are mutated. (**C**) Western blot analysis of HeLa and TRIM16$^{KO}$ lysates of cells treated with $H_2O_2$ for different durations as indicated and probed with indicated antibodies. (**D, E**) RNA isolated from cells, untreated or treated with MG132 (20 μM, 4h) for different durations and were subjected to qRT-PCR with primers of genes as indicated. The fold induction in MG132 treated samples is calculated relative to untreated samples. (**F**) Representative Confocal images of HeLa and TRIM16$^{KO}$ cells transiently expressing GFP-p62 and IF analysis is performed with Ub antibody. (**G**) Immunoprecipitation (IP) analysis of interaction between endogenous TRIM16 and endogenous LC3-II in HeLa cell lysates in absence (upper panel) and presence (lower panel) of MG132 (10 μM, 4h). *The panel H here, Fig 2 panel J and Fig EV2 panel H are part of same blots hence the input for TRIM16 is same. L.E, Low exposure; H.E, High exposure.(**H**) WB analysis of lysates from HeLa cell which were either starved or not starved in absence and presence of Bafilomycin A1 (300 nM, 3h) with the antibodies as indicated. (**I, J**) MTT assays performed at different time points with HeLa and TRIM16$^{KO}$ cells untreated or treated with $H_2O_2$ (400 μM, 2h) and MG132 (20 μM, 12h) (**K**) Immunoblot blot analysis of HeLa and TRIM16$^{KO}$ lysates of cells treated with 400 μM of $H_2O_2$ for different durations as indicated and probed with antibodies as shown.

# Appendix Supplementary Figure 4

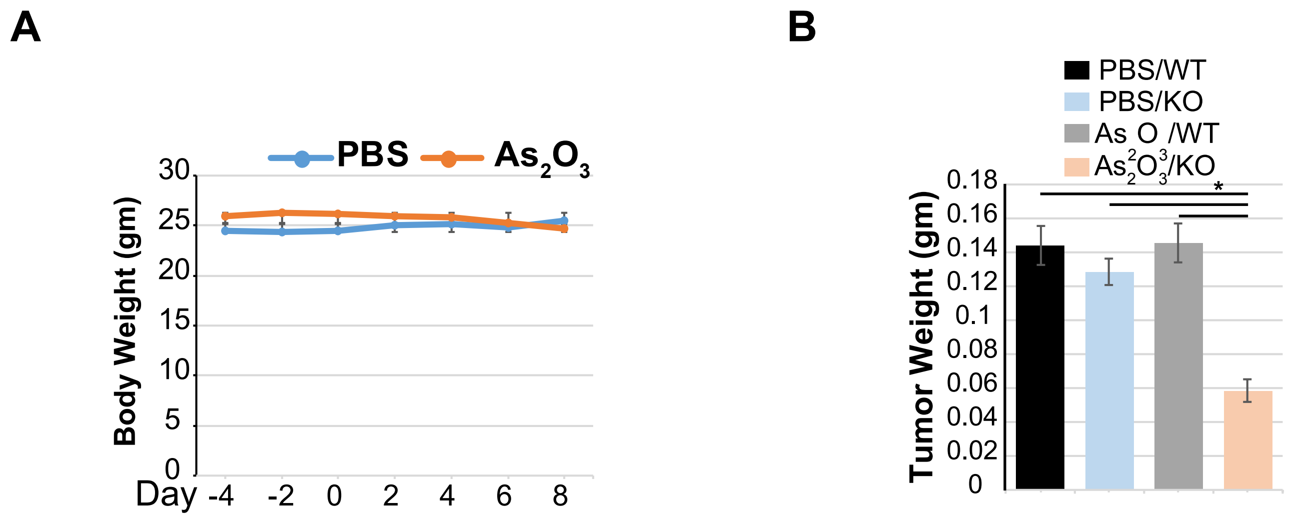


Appendix Supplementary Figure 4. (**A**) Body weight of mice throughout the experiment. (**B**) After the sacrifice of mice, tumors (n=6 for each group) were harvested and weighed (in grams). Graph, Mean ±SD, n=3, *$p < 0.05$ (ANOVA).
