# Supplementary material for: TRIM16 controls assembly and degradation of protein aggregates by modulating the p62‐NRF2 axis and autophagy
Source: EMBO J. 2018 Aug 24;37(18):e98358. doi: 10.15252/embj.201798358 (PMC6138442; doi:10.15252/embj.201798358)
Supplement: Supplementary file 2 — Expanded View Figures PDF [file EMBJ-37-e98358-s002.pdf]

Expanded View Figures

Figure EV1. TRIM16 is important for assembly of the protein aggregates.

A Confocal images of HeLa and TRIM16^{KO} cells, untreated or treated with MG132 (20 μM, 2 h), H_2O_2 (200 μM, 2 h), and puromycin (5 μg/ml, 2 h), and processed for immunofluorescence (IF) analysis with ubiquitin (FK2) antibody.

B HeLa and TRIM16^{KO} cells were visualized, and cells with more than two visible aggresomes/ALIS were considered positive. Mean ± SD, $n = 3$, $**P < 0.005$ (Student's unpaired t-test).

C Total numbers of visible Ub puncta in each cell were counted and plotted. Data are from ≥ 10 microscopic fields for each condition, $n = 3$, mean ± SD, $*P < 0.05$ (Student's unpaired t-test).

D Representative confocal images of control siRNA and TRIM16 siRNA transfected cells treated with puromycin (5 μg/ml, 2 h), where IF analysis was conducted with Ub and p62 antibodies.

E The graph shows the average number of Ub-p62 co-localized aggregates per cell from the experiment in panel (D). Mean ± SD, $n = 3$, $*P < 0.05$ (Student's unpaired t-test).

F Representative confocal images of HeLa and TRIM16^{KO} cells treated with As_2O_3 (2.5 μM, 2 h), where IF analysis was conducted with Ub and p62 antibodies. Right panel: Fluorescence intensity line is tracing corresponding to a white line in zoom panel.

G, H Representative confocal images of HeLa and TRIM16^{KO} cells treated with (G) MG132 (20 μM, 2 h) or (H) H_2O_2 (200 μM, 2 h) and processed for IF analysis with Ub and LC3B antibody.

I WB analysis of LC3B levels in lysates of cells transfected with control vector or Flag-TRIM16.

J Densitometric analysis of WB normalized with actin. Mean ± SD, $*P < 0.05$ (Student's unpaired t-test), $n = 3$.

K Representative confocal images HeLa and TRIM16^{KO} cells treated with H_2O_2 (200 μM, 2 h) and processed for IF analysis with ProteoStat dye and p62 antibody.

Data information: Unless otherwise stated, scale bar: 10 μm.
Source data are available online for this figure.

 © 2018 The Authors

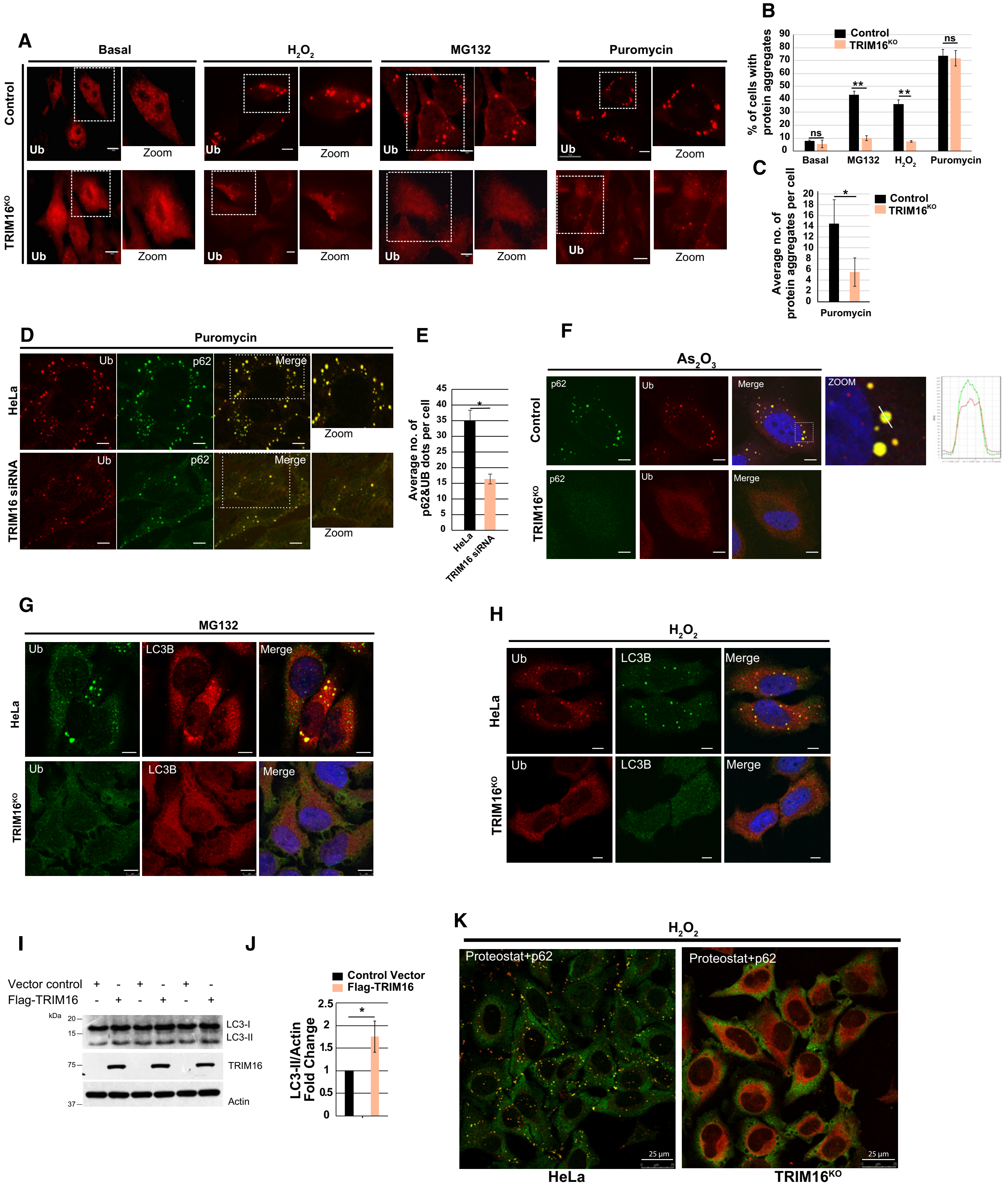
A
Basal
H2O2
MG132
Puromycin
Control
TRIM16KO
Ub
Zoom
B
% of cells with protein aggregates
Control
TRIM16KO
ns
**
Basal
MG132
H2O2
Puromycin
C
Average no. of protein aggregates per cell
Puromycin
D
Puromycin
HeLa
TRIM16 siRNA
Ub
p62
Merge
Zoom
E
Average no. of p62&UB dots per cell
HeLa
TRIM16 siRNA
F
As2O3
Control
TRIM16KO
p62
Ub
Merge
ZOOM
G
MG132
HeLa
TRIM16KO
Ub
LC3B
Merge
H
H2O2
HeLa
TRIM16KO
Ub
LC3B
Merge
I
Vector control + - + - + -
Flag-TRIM16 - + - + - +
kDa
LC3-I
LC3-II
TRIM16
Actin
J
Control Vector
Flag-TRIM16
LC3-II/Actin Fold Change
K
H2O2
Proteostat+p62
25 µm
HeLa
TRIM16KO

Figure EV1.

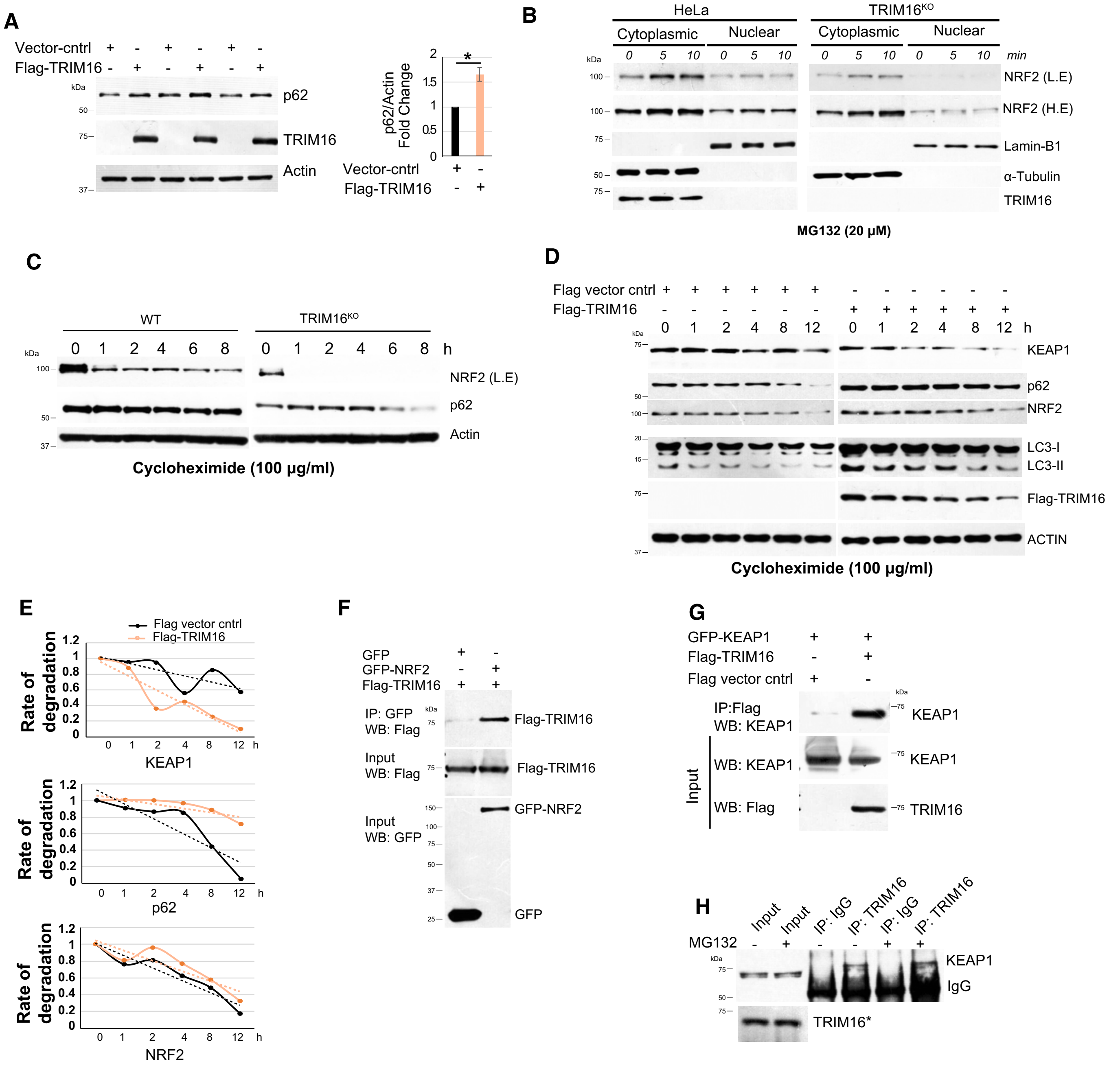

Figure EV2. TRIM16 interacts with and regulates the expression of p62, KEAP1, and NRF2.

A Left panel, HEK293T cells were either transiently transfected with an empty vector or a Flag-tagged-TRIM16 expressing vector and immunoblotted with Flag or indicated antibodies. Right panel, densitometric analysis (mean ± SD) of protein band intensity relative to actin, $n = 3$, $*P < 0.05$ (Student's unpaired t-test).

B Western blot analysis of cytoplasmic and nuclear fraction isolated from MG132-treated control and TRIM16^{KO} cells.

C WB analysis of HeLa and TRIM16^{KO} lysates of cells treated with cycloheximide (100 μg/ml) for the indicated period and probed with different antibodies as indicated. The blots of control and TRIM16^{KO} cells are exposed for the equal duration and developed together on the same X-ray film.

D WB analysis of control or Flag-TRIM16 vector transfected HeLa cells treated with cycloheximide (100 μg/ml) for the indicated period and probed with different antibodies as indicated. The blots of control and overexpressed cells are exposed for the equal duration and developed together on the same X-ray film.

E Quantification of KEAP1, p62, and NRF2 band intensities (from panel D) relative to actin.

F Co-IP analysis of the interaction between TRIM16 and NRF2 in HEK293T lysates of cells expressing GFP or GFP-NRF2 and Flag-TRIM16.

G Co-IP analysis of the interaction between TRIM16 and KEAP1 in HEK293T lysates of cells expressing vector control or Flag-TRIM16 and GFP-KEAP1.

H IP analysis of the interaction between endogenous TRIM16 and endogenous KEAP1 in HeLa cell lysates in absence and presence of MG132. *The panel (H) here, Fig 2J, and Appendix Fig S3H are part of same blots; hence, the input for TRIM16 is same.

Source data are available online for this figure.

 © 2018 The Authors

Figure EV3. TRIM16 regulates ubiquitination of NRF2 and governs the NRF2 stress responses.

A Representative confocal images of HeLa, TRIM16^{KO} cells, and TRIM16^{KO} cells complemented with TRIM16 deletion constructs where cells were treated with MG132 (20 μM, 2 h) and the samples were processed for IF analysis with Ub antibody.
B The graph shows the percentage of cells with Ub-positive protein aggregates. Data from ≥ 10 fields (40×), n = 3, mean ± SD, *P < 0.005 (Student's unpaired t-test).
C HeLa and TRIM16^{KO} cells were treated with MG132 (10 μM, 4 h), and lysates were subjected to Western blotting with indicated antibodies.
D WB analysis of HeLa and TRIM16^{KO} lysates of cells treated with MG132 (10 μM, 4 h) or/and BafA1 (300 nM, 4 h) as indicated and probed with antibodies as shown.
E HeLa and TRIM16^{KO} cells were treated with MG132 (10 μM, 4 h), and lysates were subjected to Western blotting with indicated antibodies.
F Quantification of UB lane intensity relative to actin. Mean ± SD, n = 3, *P < 0.05.
G HeLa and TRIM16^{KO} cells were treated with MG132 (10 μM, 4 h), and lysates were subjected to Western blotting with K48 and K63 and other indicated antibodies.
H–J RNA isolated from HeLa and TRIM16^{KO} cells, untreated or treated with MG132 (20 μM, 2 h), were subjected to qRT–PCR with primers of genes as indicated. The fold induction in MG132-treated samples is calculated relative to untreated samples. Mean ± SD, n = 3, *P < 0.05, #Insignificant.

Data information: Unless otherwise stated, scale bar: 10 μm.

Source data are available online for this figure.

© 2018 The Authors

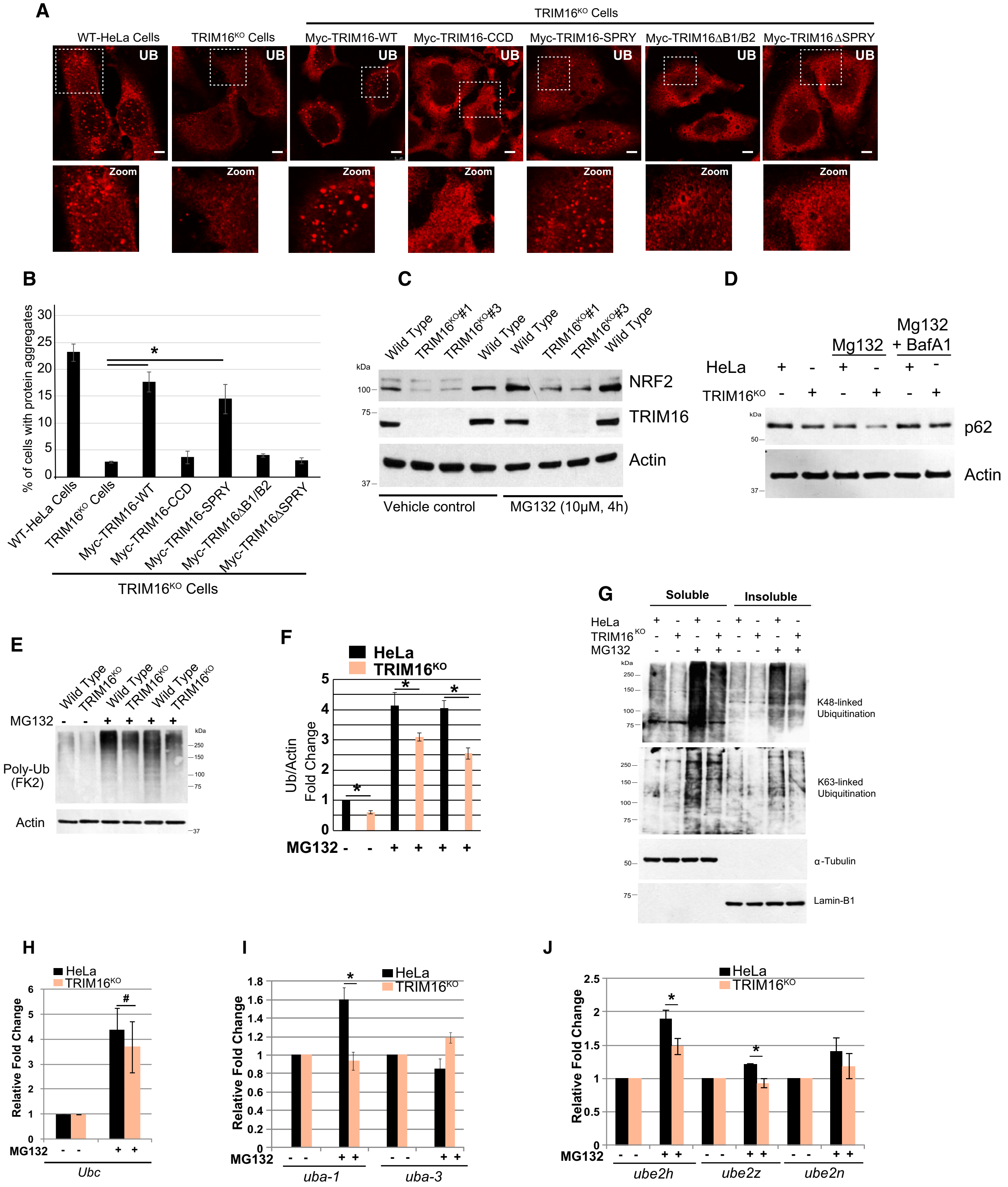

Figure EV3.

© 2018 The Authors

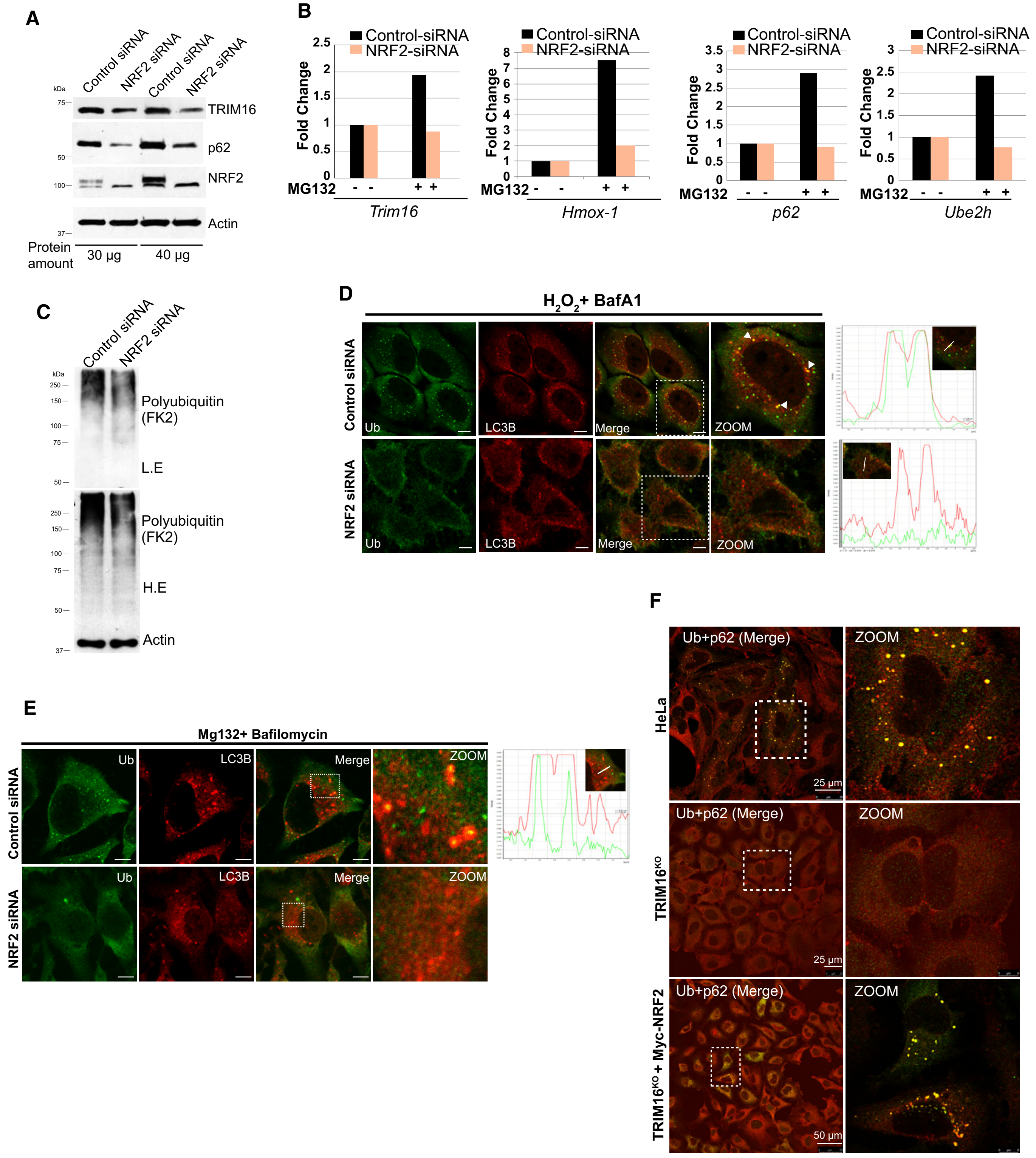
A
Control siRNA
NRF2 siRNA
Control siRNA
NRF2 siRNA
kDa
TRIM16
p62
NRF2
Actin
Protein amount
30 µg
40 µg
B
Control-siRNA
NRF2-siRNA
Fold Change
MG132
Trim16
Hmox-1
p62
Ube2h
C
Polyubiquitin (FK2)
L.E
H.E
Actin
D
H2O2+ BafA1
Control siRNA
NRF2 siRNA
Ub
LC3B
Merge
ZOOM
E
Mg132+ Bafilomycin
F
Ub+p62 (Merge)
HeLa
TRIM16KO
TRIM16KO+ Myc-NRF2
25 µm
50 µm

Figure EV4

Figure EV4. NRF2 regulates TRIM16, ubiquitin pathway genes expression, and biogenesis of protein aggregates.

A Control or NRF2 siRNA transfected HeLa cells lysates were subjected to Western blotting with indicated antibodies (different protein concentrations as indicated).

B RNA isolated from untreated or MG132-treated control (20 μM, 2 h) or NRF2 siRNA transfected cells were subjected to qRT–PCR with primers of genes as indicated. The fold induction in MG132-treated samples is calculated relative to untreated samples.

C WB analysis of control siRNA or NRF2 siRNA transfected lysates of HeLa cells probed with antibodies as shown.

D Left panels: Confocal images of control and NRF2 siRNA transfected cells treated with H_2O_2 (200 μM, 2 h) where IF analysis was conducted with Ub and LC3B antibodies. Right panels: Fluorescence intensity line is tracing corresponding to a white line in zoom panel. Arrowheads indicate the puncta where co-localization between UB and LC3B is observed.

E Representative confocal images of control and NRF2 siRNA transfected cells treated with MG132 (20 μM, 2 h) and bafilomycin A1 (300 nM, 3 h) and IF analysis was conducted with Ub and LC3B antibodies. Right panels: fluorescence intensity line is tracing corresponding to the white line (inset).

F Representative confocal images of HeLa cells, $TRIM16^{KO}$ cells, and $TRIM16^{KO}$ cells complemented with Myc-NRF2 were treated with MG132 (20 μM, 2 h) and subjected to IF with Ub and p62 antibodies.

Data information: Unless otherwise stated, scale bar: 10 μm.

Source data are available online for this figure.

 © 2018 The Authors

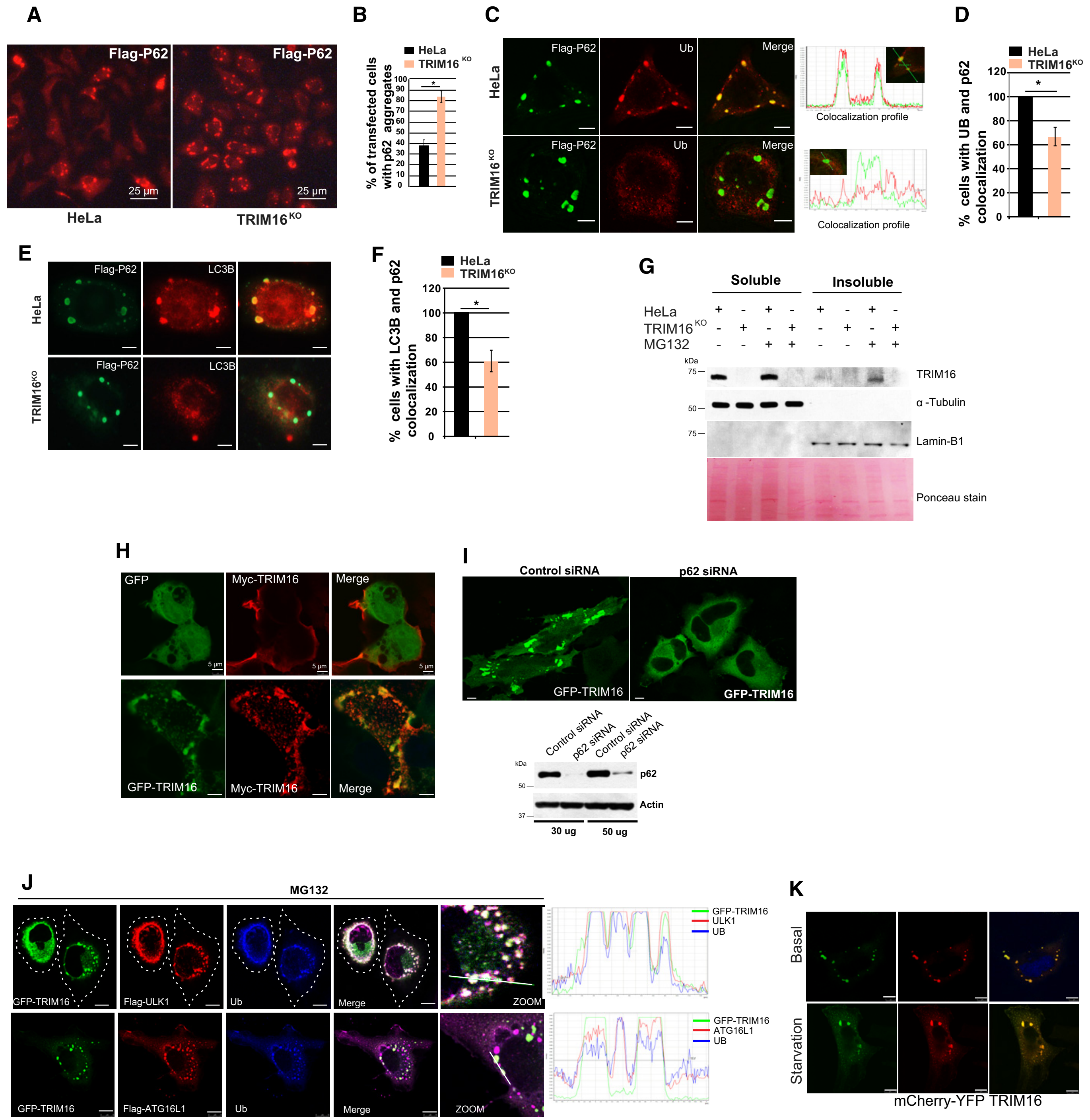
A
Flag-P62
Flag-P62
25 µm
25 µm
HeLa
TRIM16KO
B
HeLa
TRIM16KO
% of transfected cells with p62 aggregates
C
HeLa
TRIM16KO
Flag-P62
Ub
Merge
Colocalization profile
D
% cells with UB and p62 colocalization
E
Flag-P62
LC3B
F
% cells with LC3B and p62 colocalization
G
Soluble
Insoluble
HeLa
TRIM16KO
MG132
kDa
TRIM16
α -Tubulin
Lamin-B1
Ponceau stain
H
GFP
Myc-TRIM16
Merge
GFP-TRIM16
I
Control siRNA
p62 siRNA
GFP-TRIM16
p62
Actin
30 ug
50 ug
J
MG132
GFP-TRIM16
Flag-ULK1
Ub
Merge
ZOOM
Flag-ATG16L1
ULK1
UB
ATG16L1
K
Basal
Starvation
mCherry-YFP TRIM16

Figure EV5.

Figure EV5. TRIM16 mediates selective clearance of aggregated autophagy cargoes.

A Representative confocal images of HeLa and $TRIM16^{KO}$ cells transiently expressing Flag-p62.

B The graph shows the percentage of transfected cells with p62-aggregates. Data from ≥ 10 microscopic fields for each condition (40×), $n = 3$, mean ± SD, *$P < 0.05$ (Student's unpaired t-test).

C–F (C, E) Representative confocal images of HeLa and $TRIM16^{KO}$ cells expressing Flag-p62 and IF analysis was performed with Flag and (C) Ub or (E) LC3B antibodies. (D, F) The graph shows the percentage of cells with p62-Ub (D) or p62-LC3B (F) co-localized aggregates. Data from ≥ 10 microscopic fields, $n = 3$, mean ± SD, *$P < 0.05$ (Student's unpaired t-test).

G WB analysis of detergent-soluble and detergent-insoluble fractions of HeLa and $TRIM16^{KO}$ cells, untreated or treated with MG132 (10 μM, 4 h), and probed with indicated antibodies.

H Representative confocal images of HEK293T cells transiently expressing GFP-TRIM16 and Myc-TRIM16.

I Upper panel, representative confocal images of control or p62 siRNA transfected HeLa cells transiently expressing GFP-TRIM16. Lower panel, WB showing the knockdown efficiency of p62 siRNA.

J Representative confocal images of HeLa cells expressing GFP-TRIM16, Flag-ULK1, or Flag-ATG16L1 treated with MG132 (10 μM, 4 h) and/or bafilomycin A1 (300 nM, 3 h) and IF analysis is performed with antibodies as indicated. Right: Fluorescence intensity line is tracing corresponding to the white line.

K Representative confocal images of starved and unstarved HEK293T cells transiently expressing mcherry-YFP-TRIM16.

Data information: Unless otherwise stated, scale bar: 10 μm.

Source data are available online for this figure.

© 2018 The Authors
