# Supplementary material for: TRIM16 controls assembly and degradation of protein aggregates by modulating the p62‐NRF2 axis and autophagy
Source: EMBO J. 2018 Aug 24;37(18):e98358. doi: 10.15252/embj.201798358 (PMC6138442; doi:10.15252/embj.201798358)

Fig EV1

Panel I

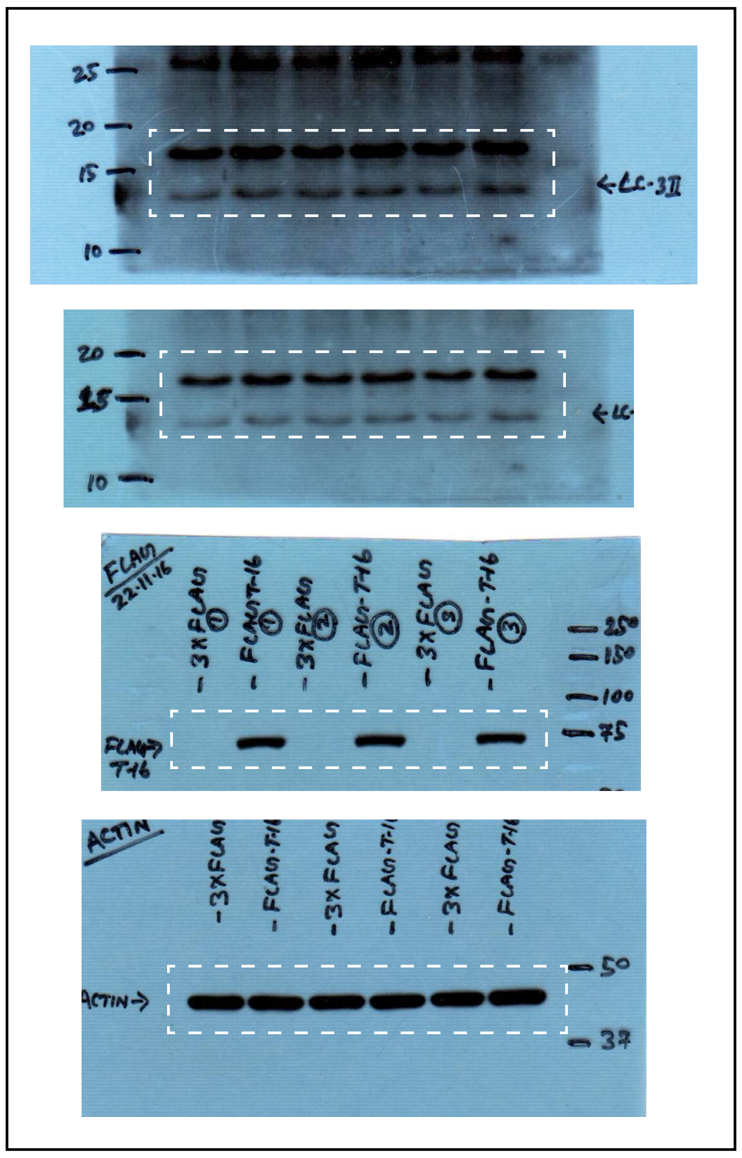

Fig EV2

Panel A

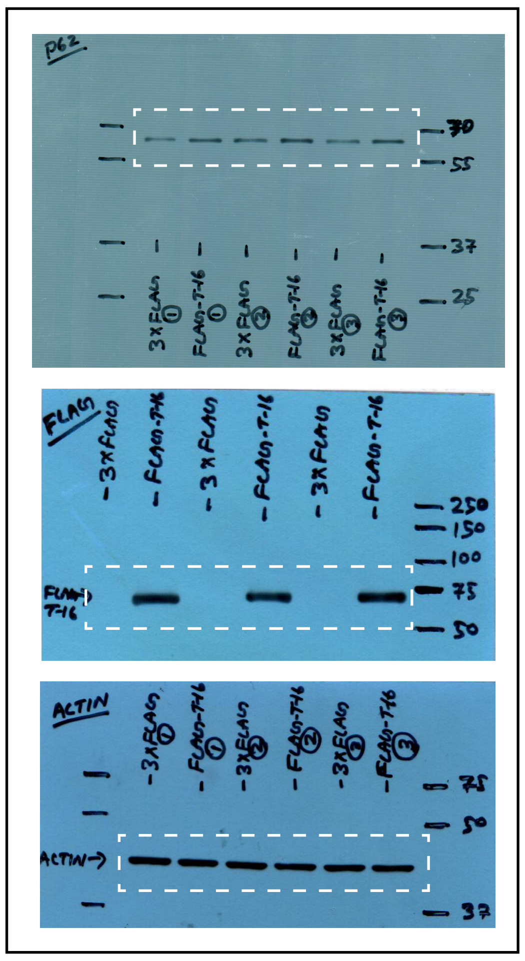

Panel B

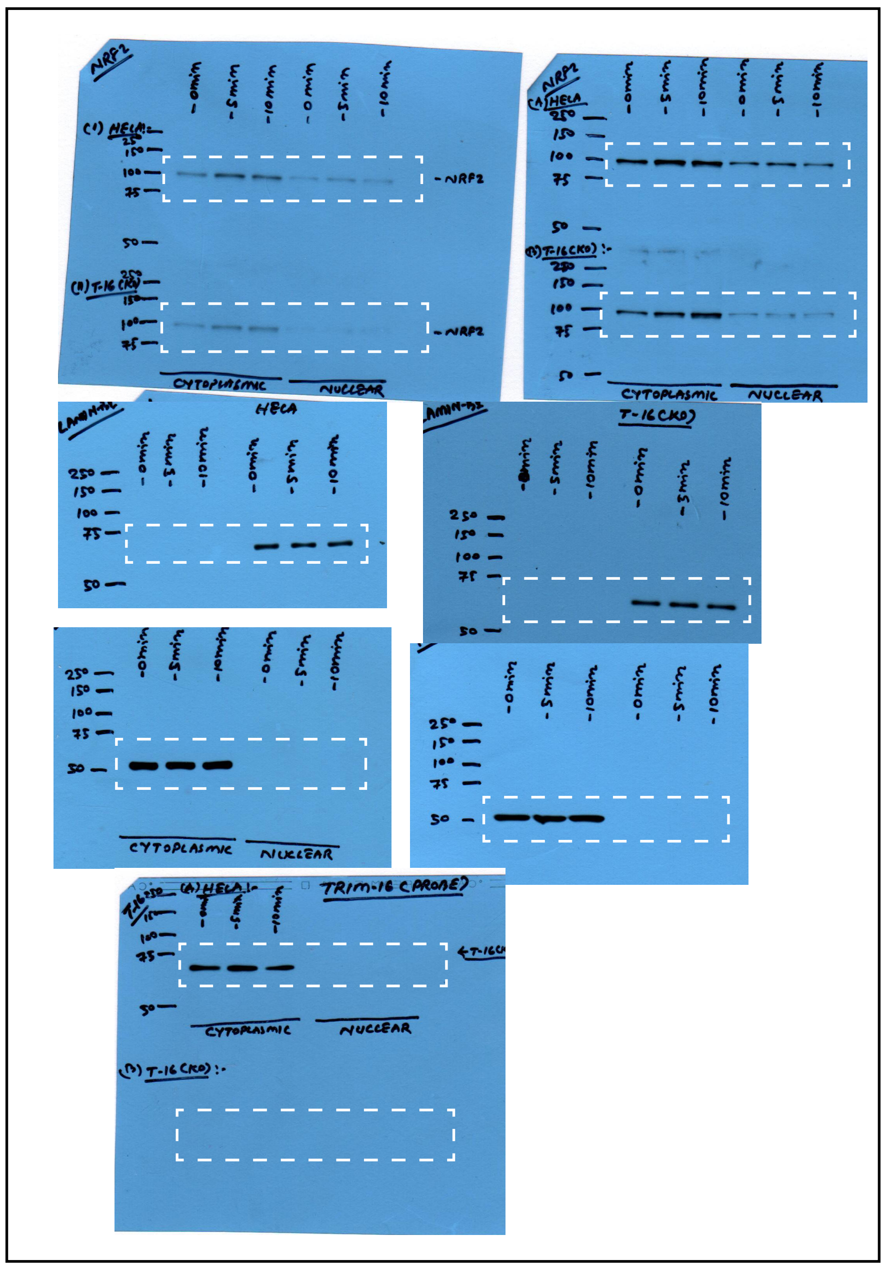

Panel C

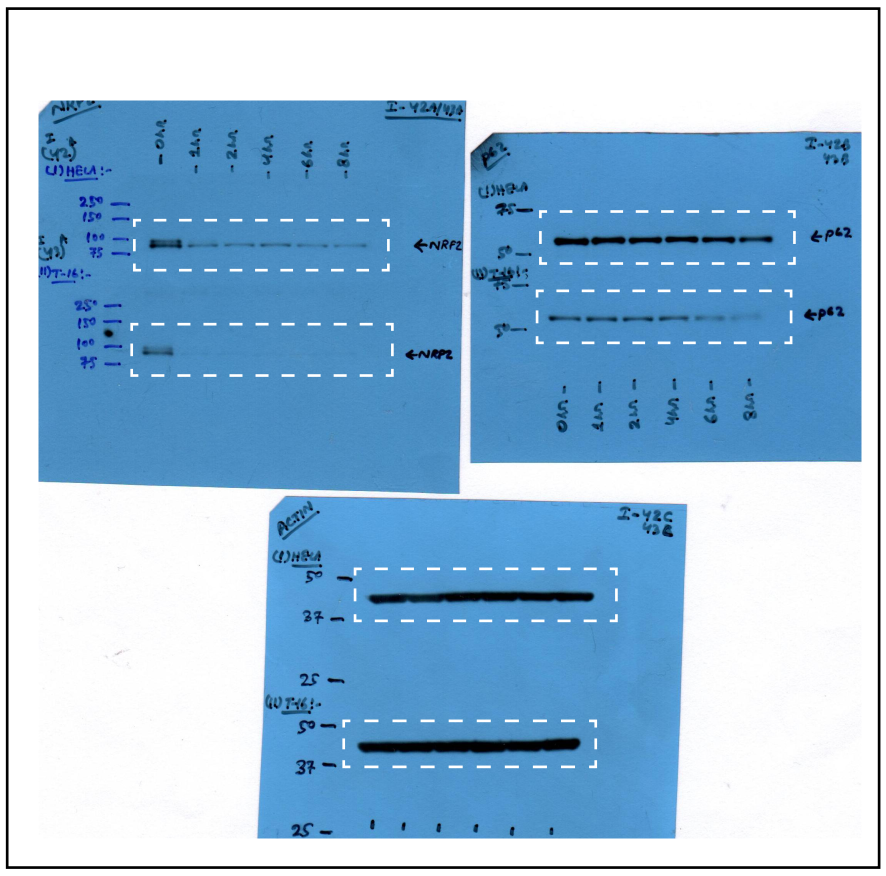

Panel D

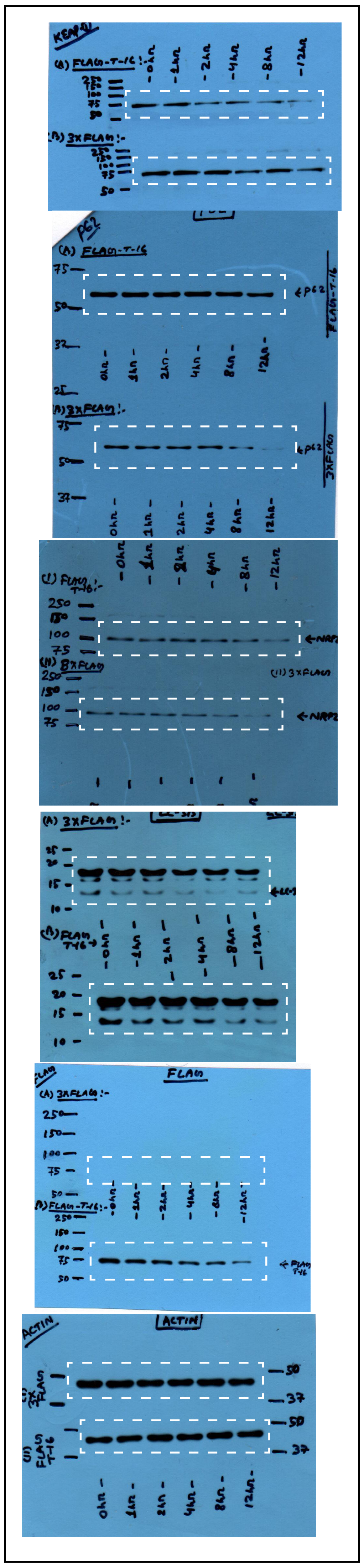

Panel F

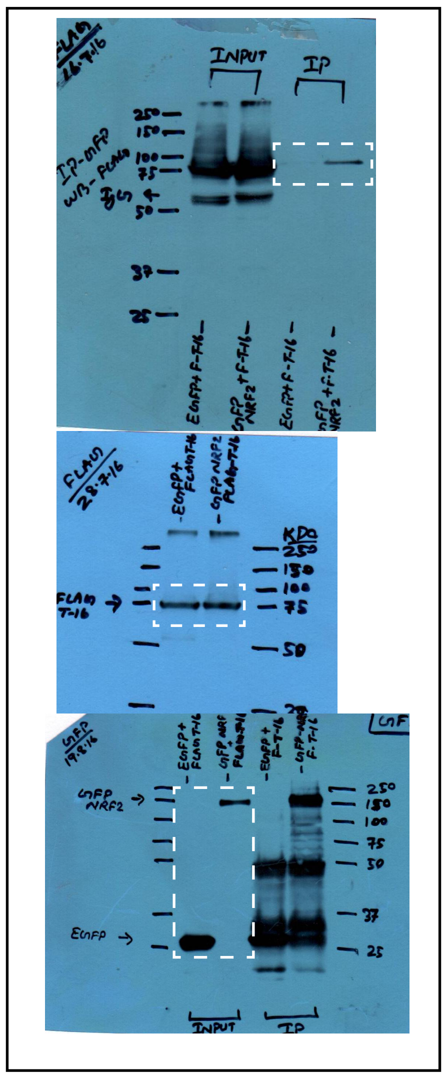

Panel G

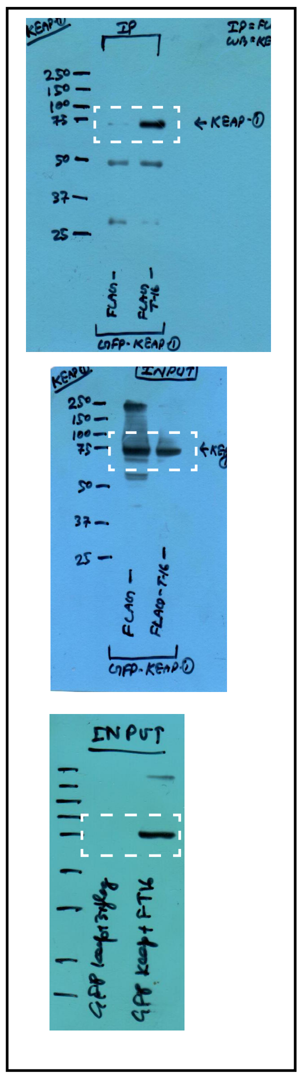

Panel H

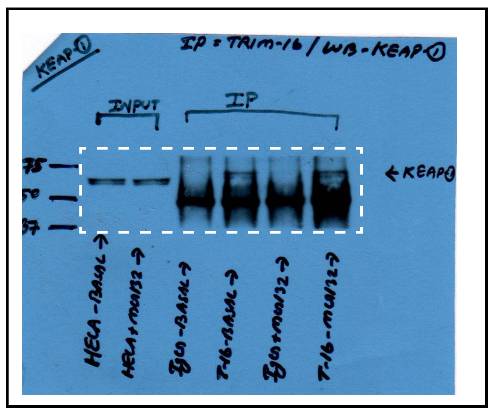

Fig EV3

Panel C

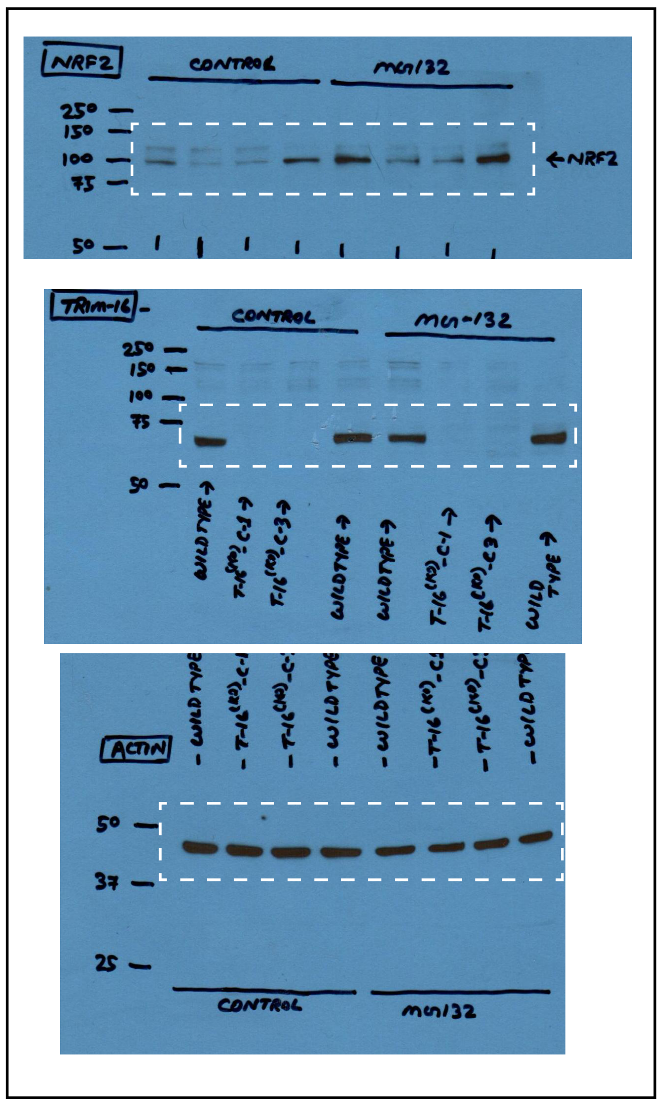

Panel D

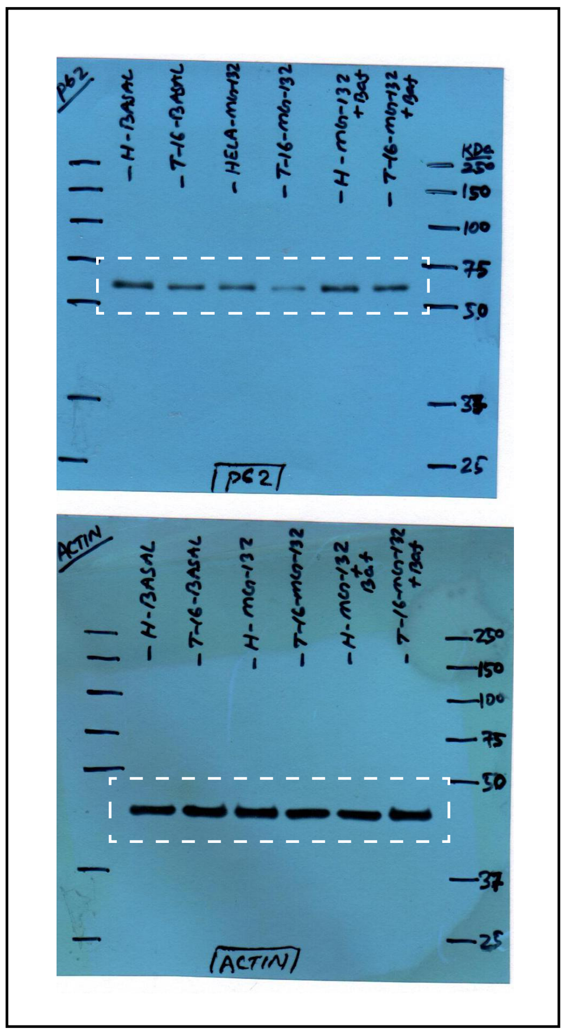

Panel E

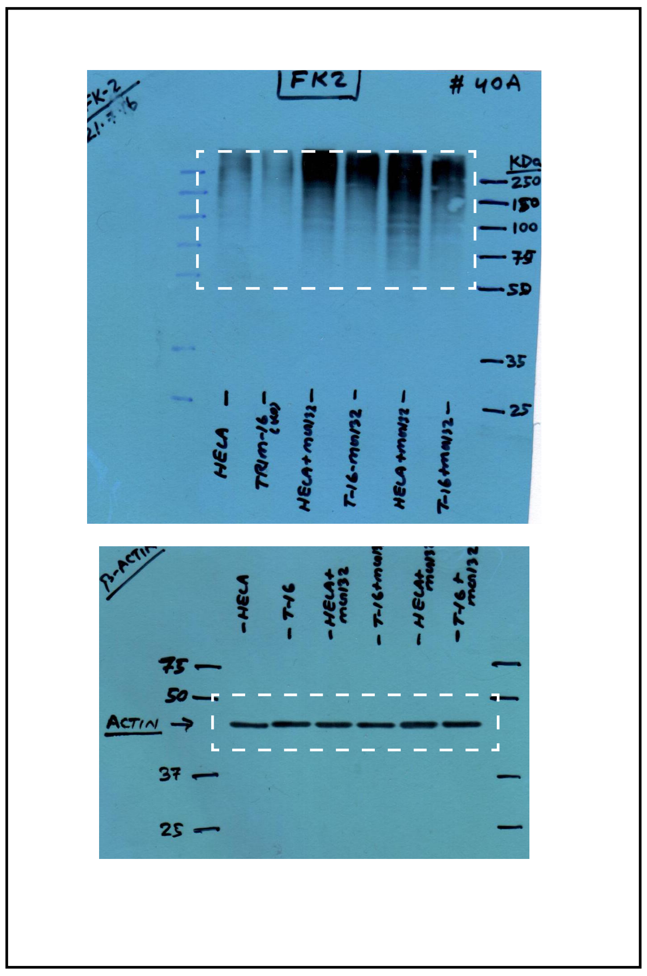

Panel G

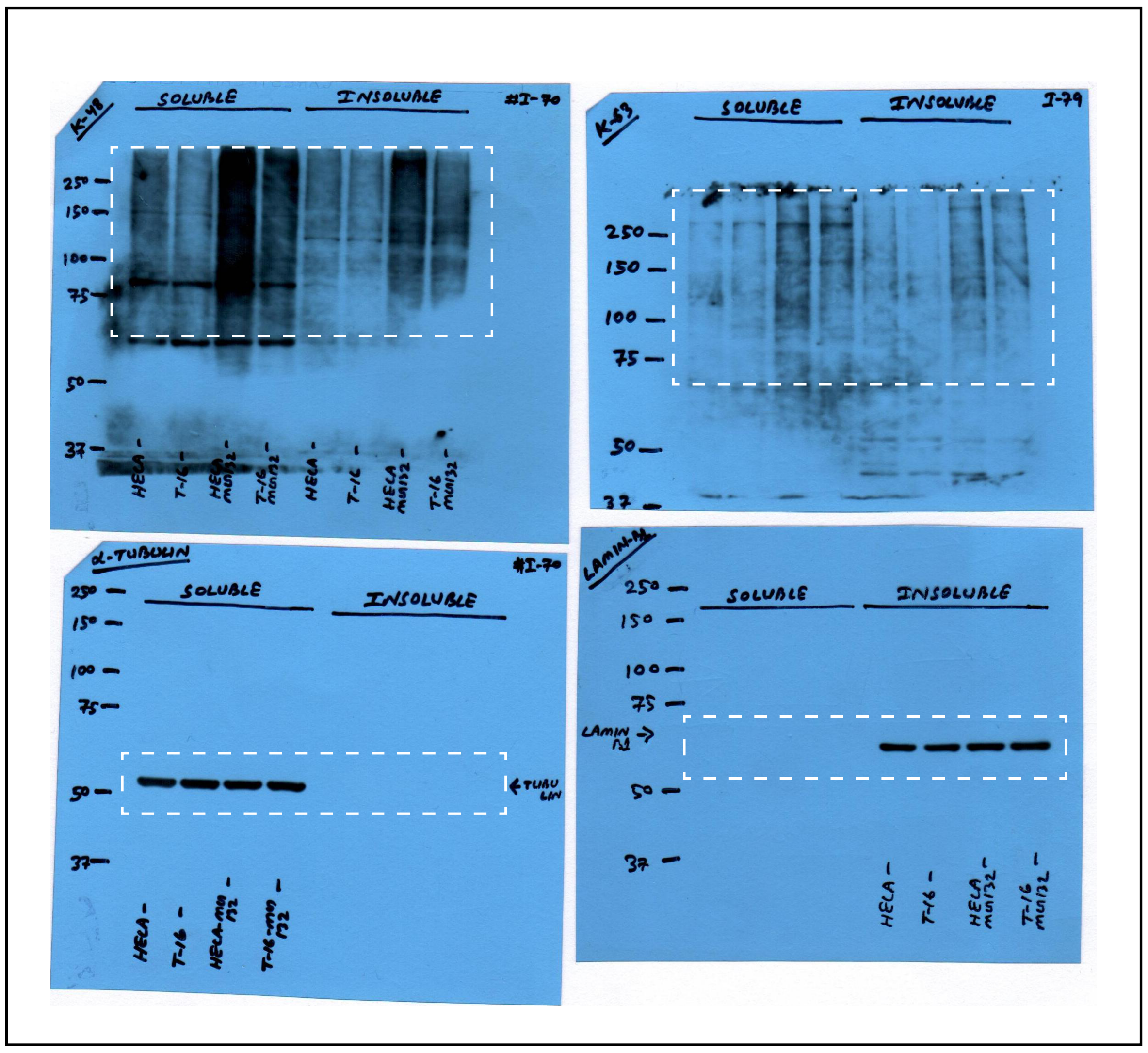

Fig EV4

Panel A

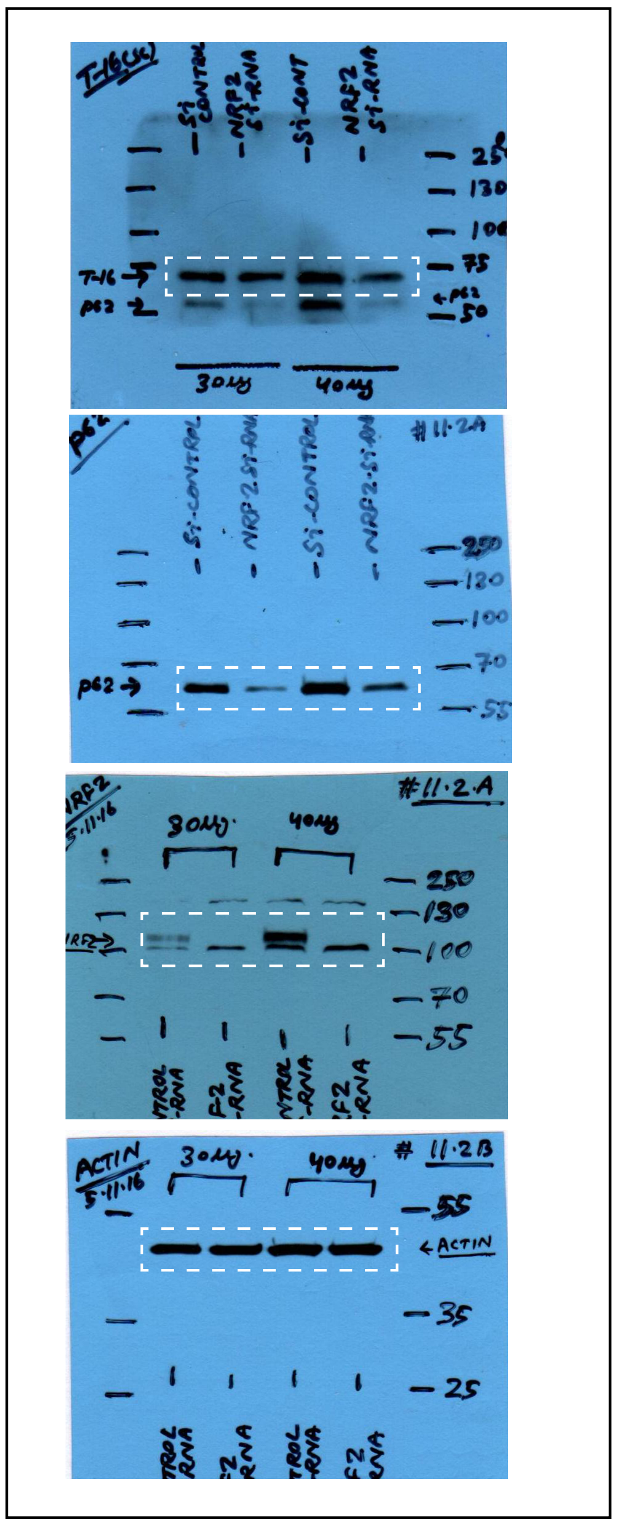

Panel C

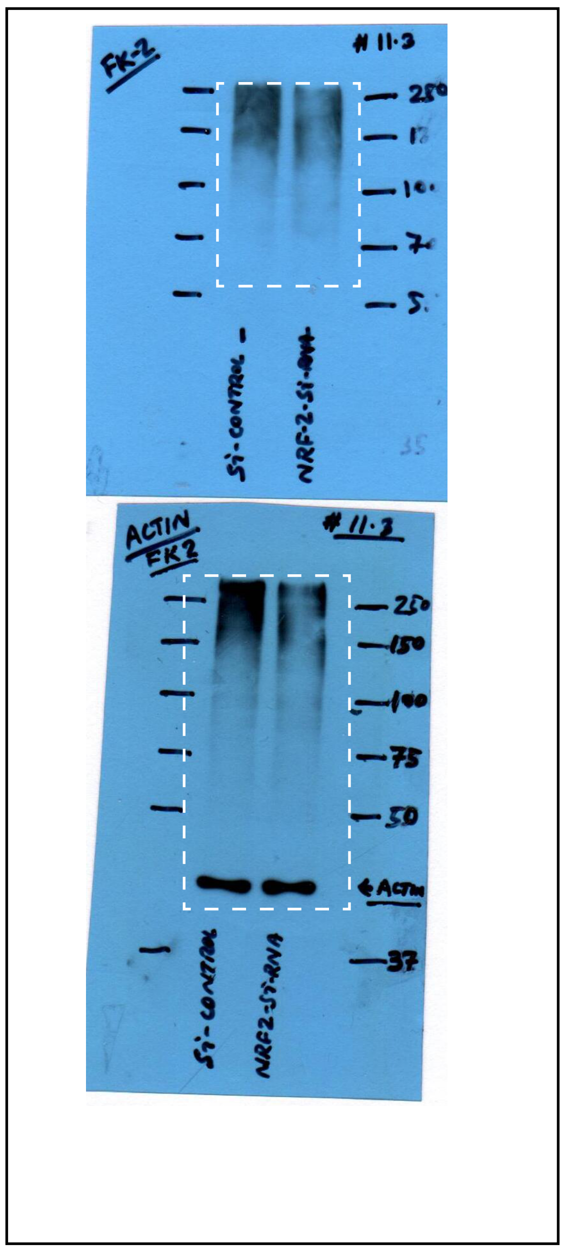

Fig EV5

Panel G

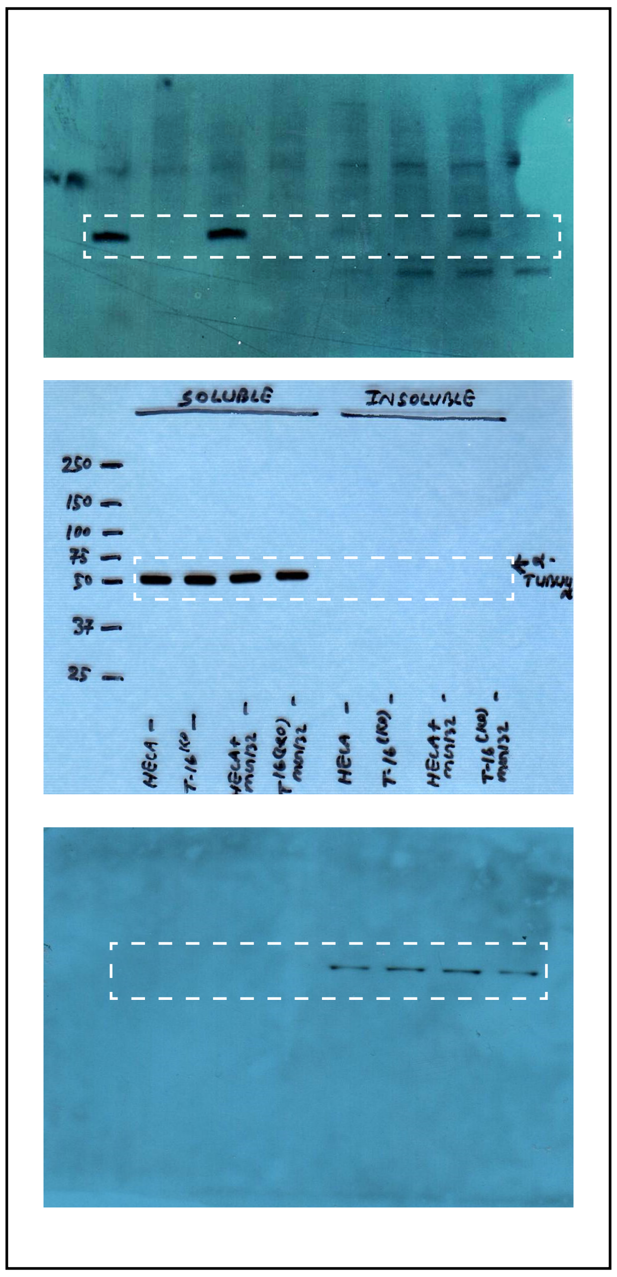

Panel I

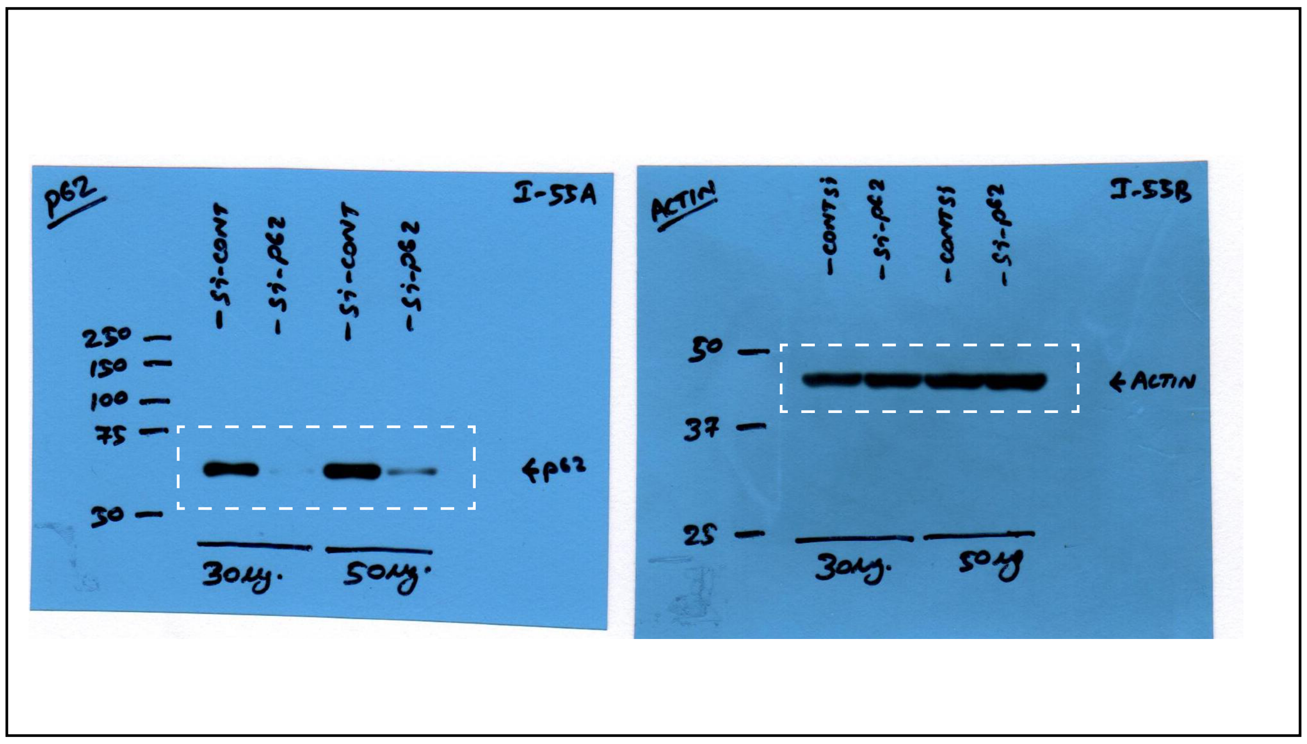

Supplement: Supplementary file 3 — Source Data for Expanded View [file EMBJ-37-e98358-s012.zip › Source_Data_for_EV_Figures/Source_Data_for_EV_Figures.pdf]
