# Supplementary figures and images for: TRIM16 controls assembly and degradation of protein aggregates by modulating the p62‐NRF2 axis and autophagy
Source: EMBO J. 2018 Aug 24;37(18):e98358. doi: 10.15252/embj.201798358 (PMC6138442; doi:10.15252/embj.201798358)

Figure-1

Panel G

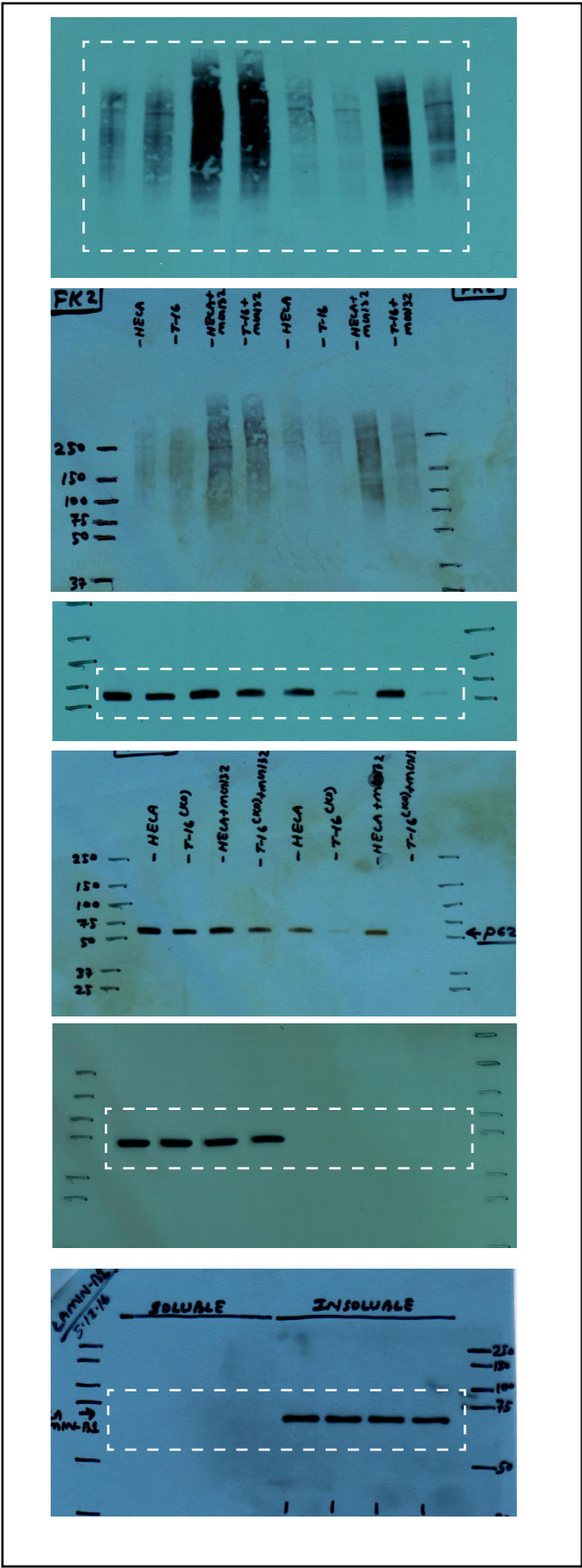

Panel K

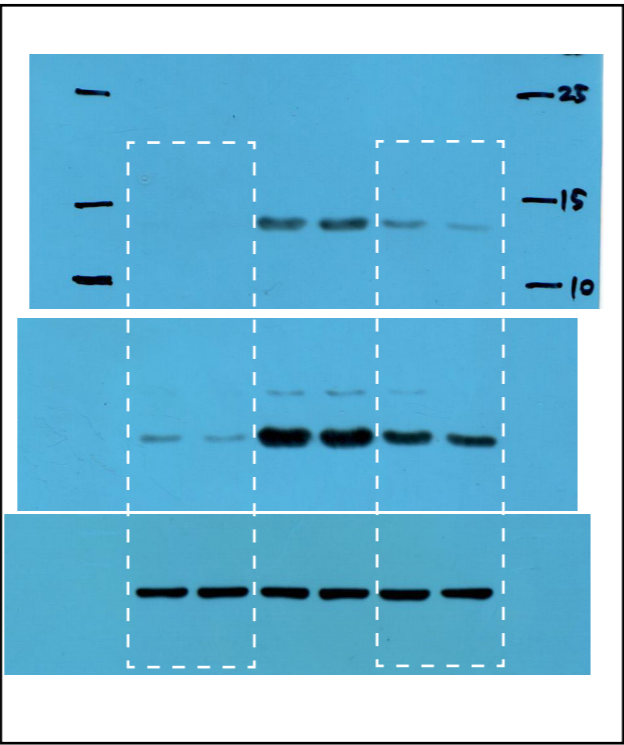

Panel L

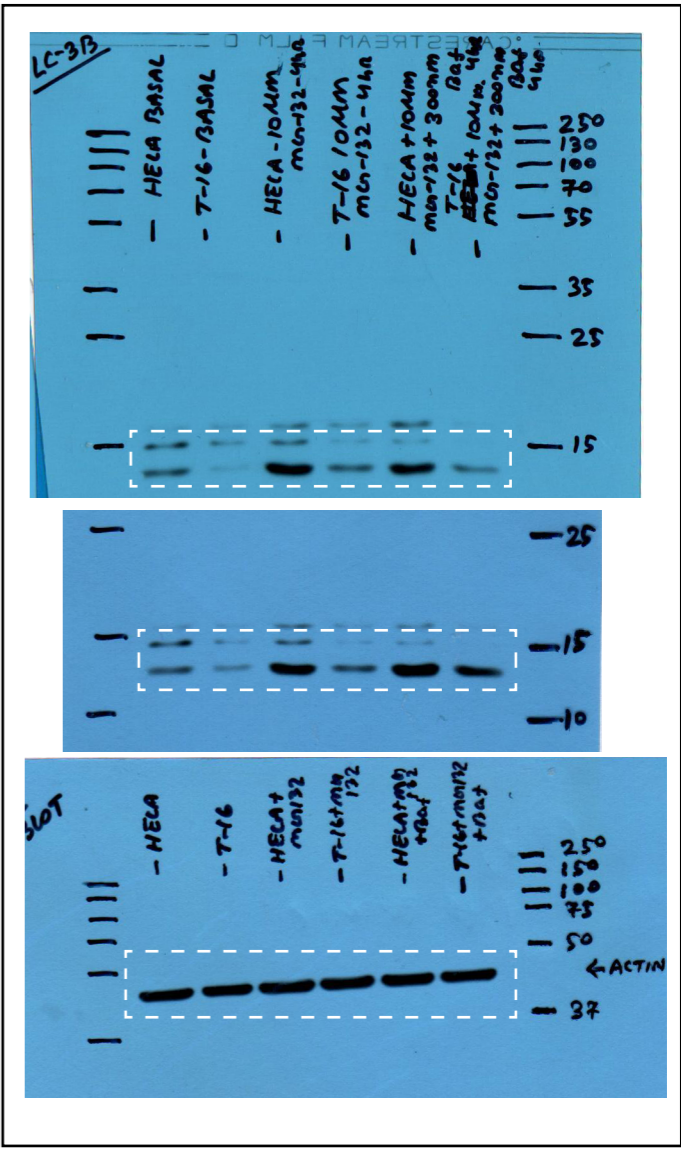

Supplement: Supplementary file 5 — Source Data for Figure 1 [file EMBJ-37-e98358-s003.pdf]

Figure 2

Panel A

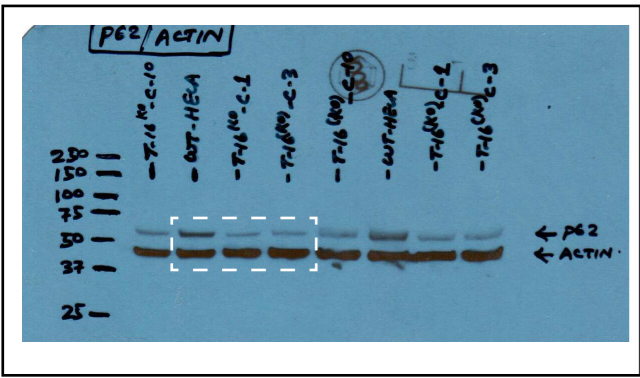

Panel B

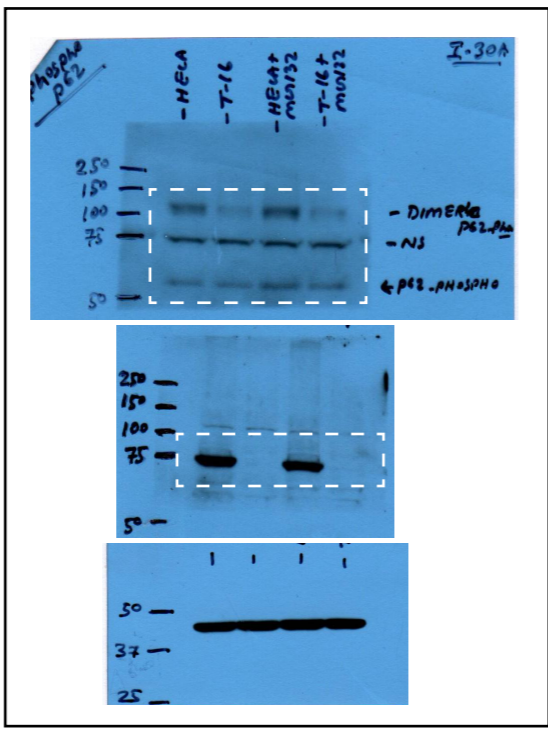

Panel C

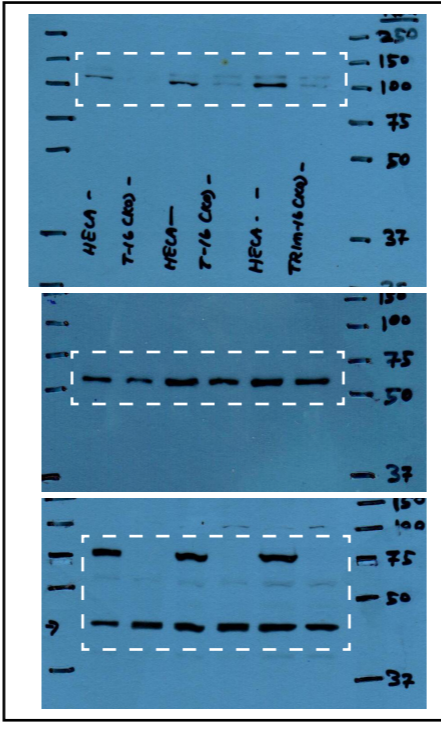

Panel D

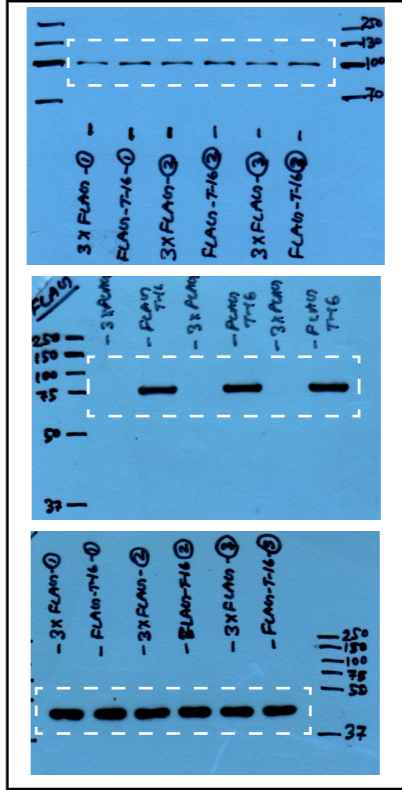

Panel F

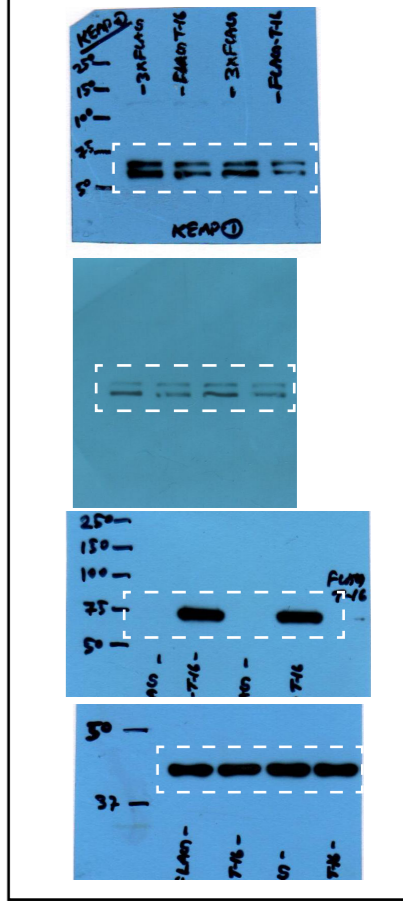

Panel H

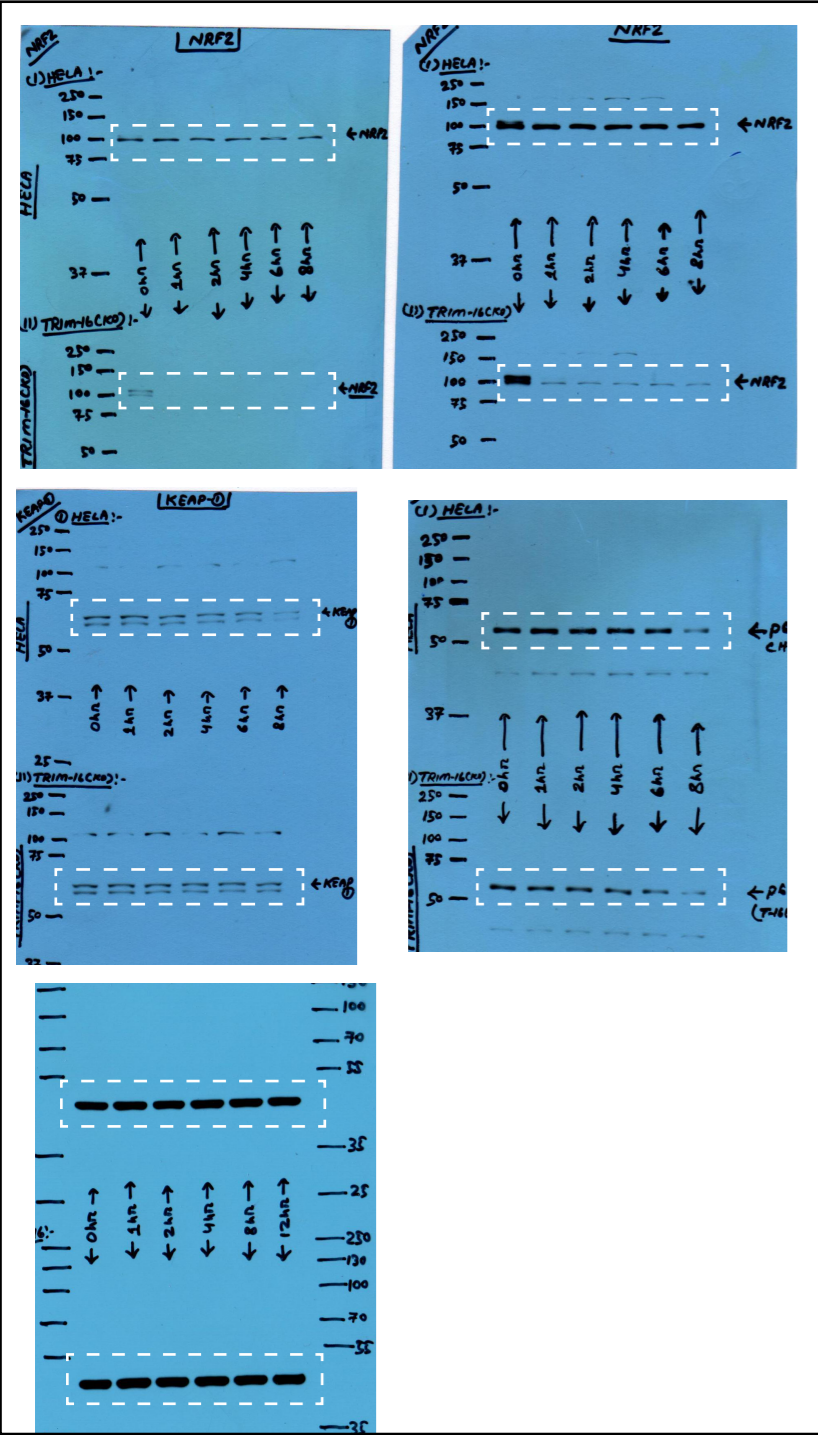

Panel J

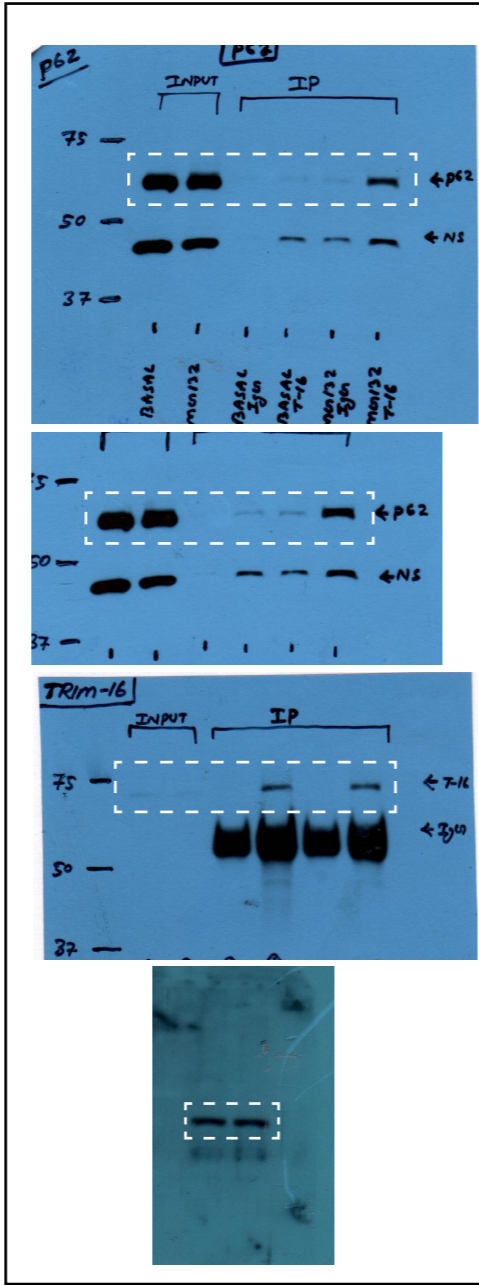

Panel L

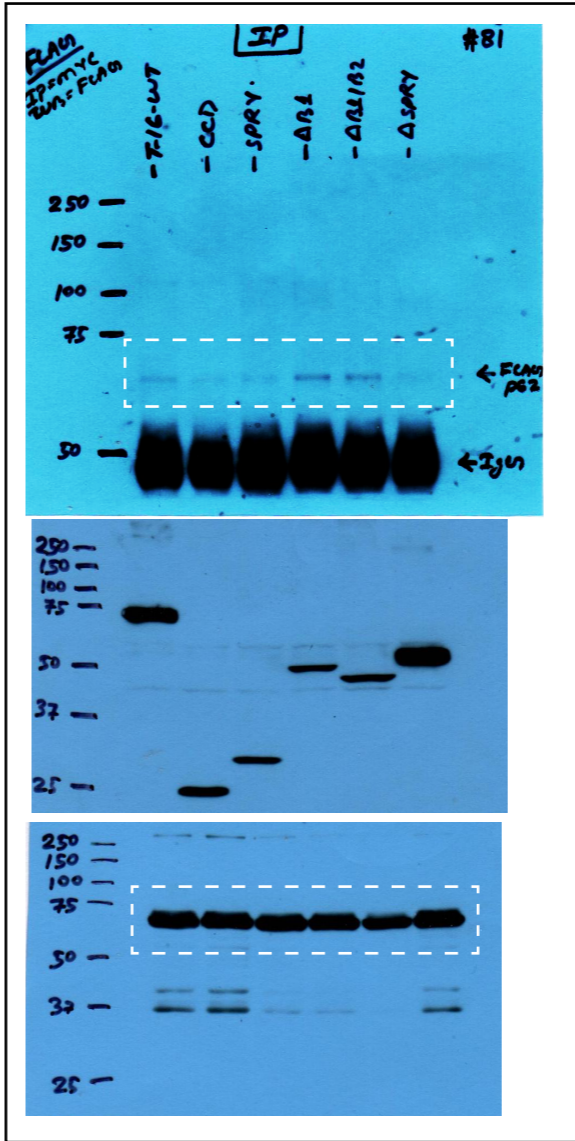

Panel M

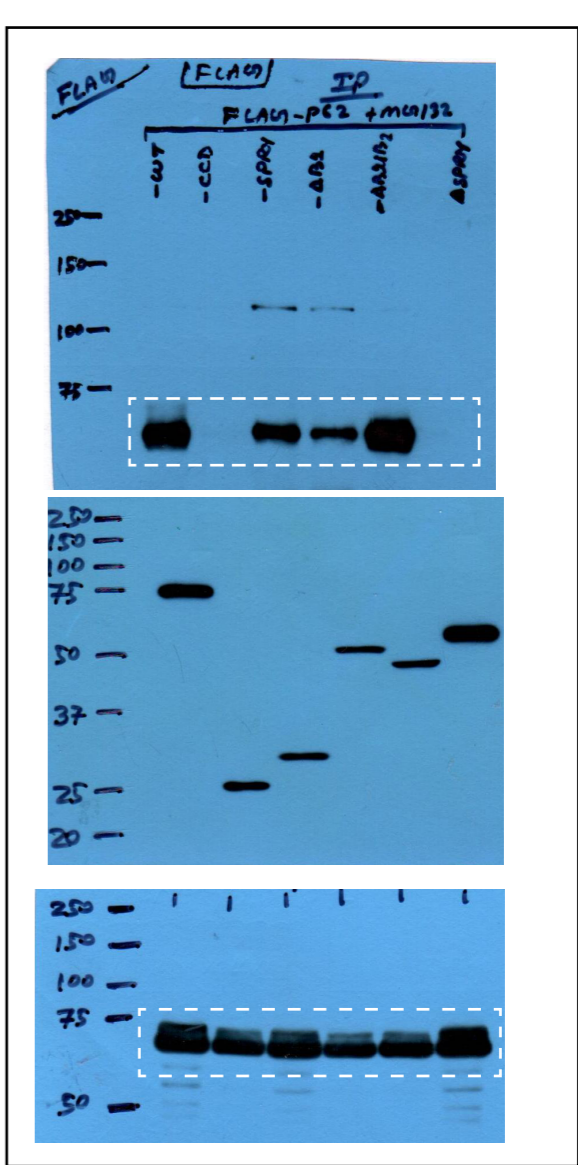

Panel N

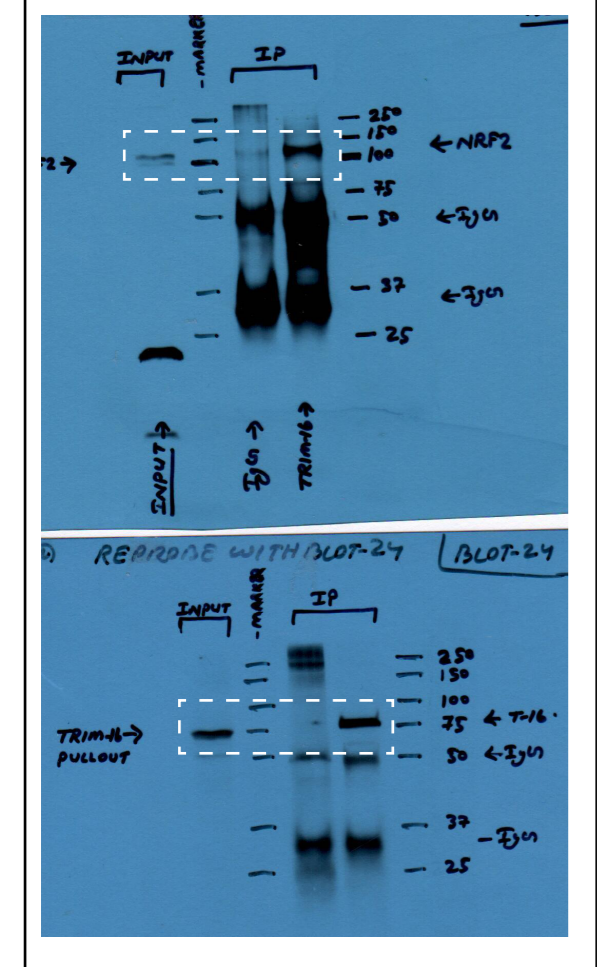

Panel O

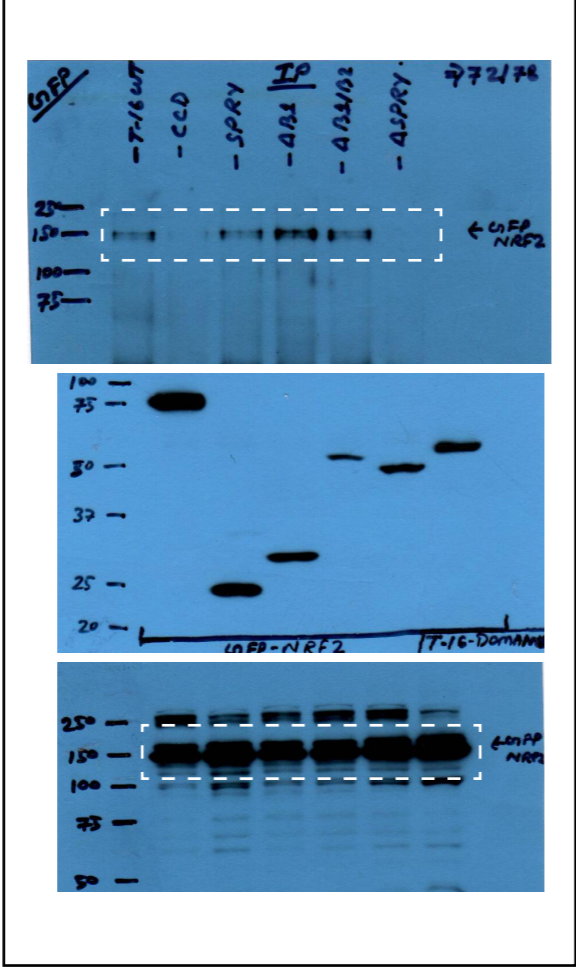

Panel P

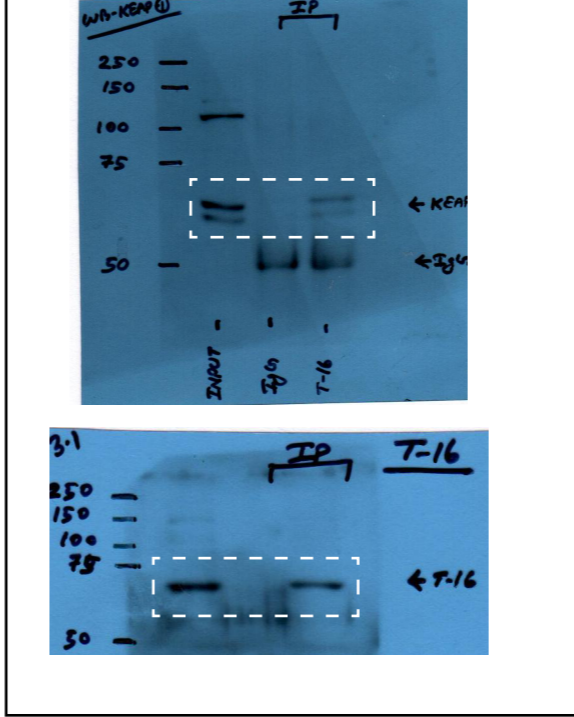

Supplement: Supplementary file 6 — Source Data for Figure 2 [file EMBJ-37-e98358-s004.pdf]

Figure 3

Panel A

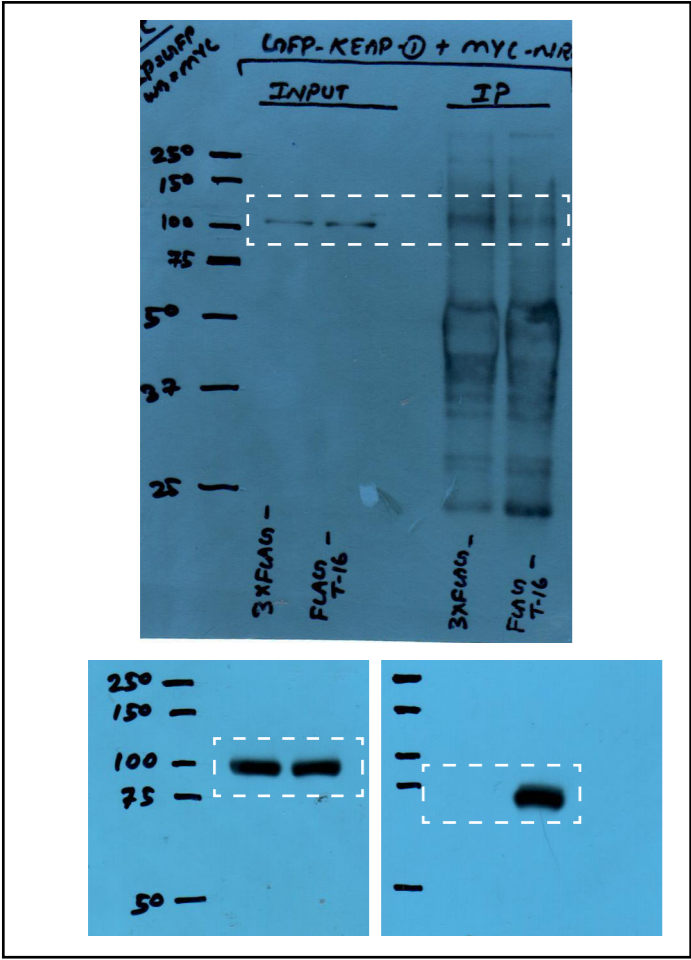

Panel B

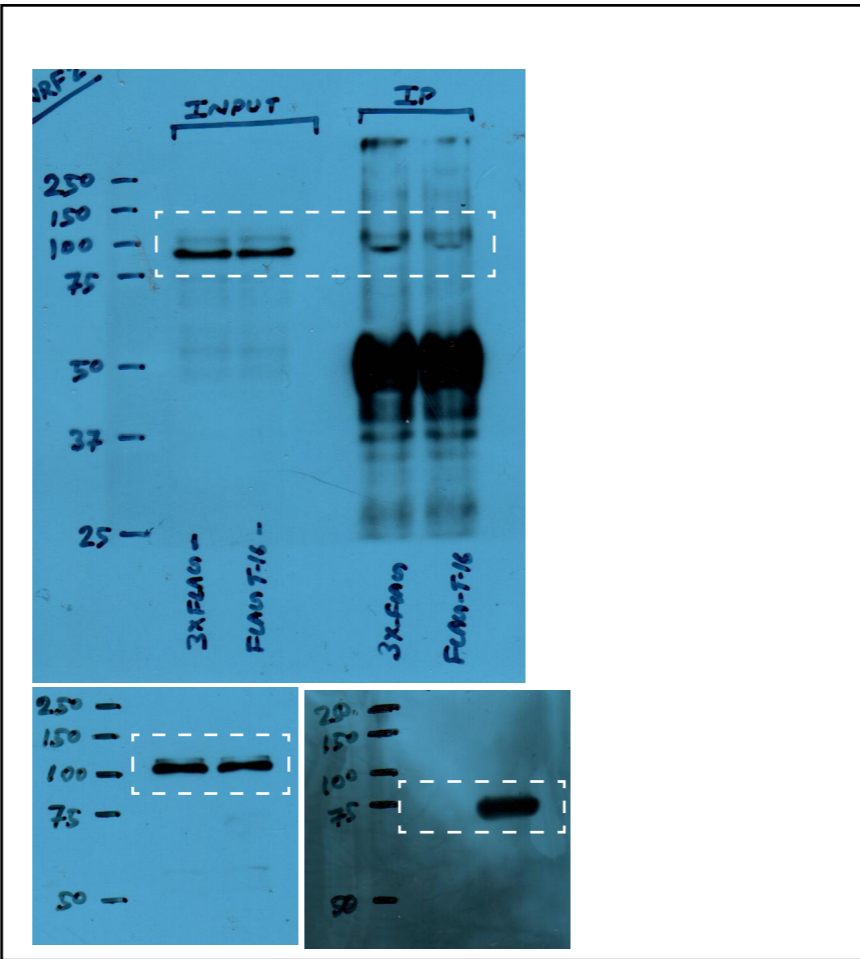

Panel C

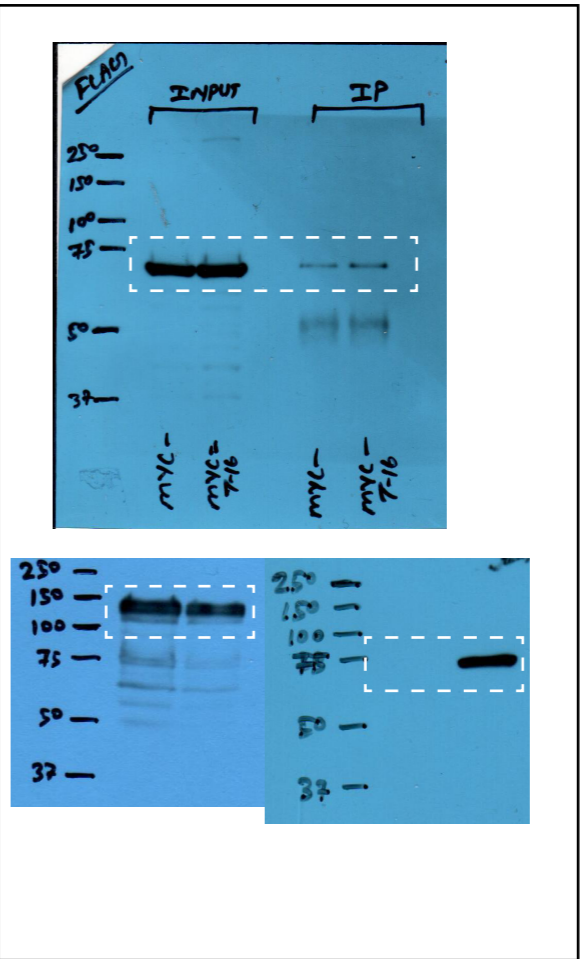

Panel D

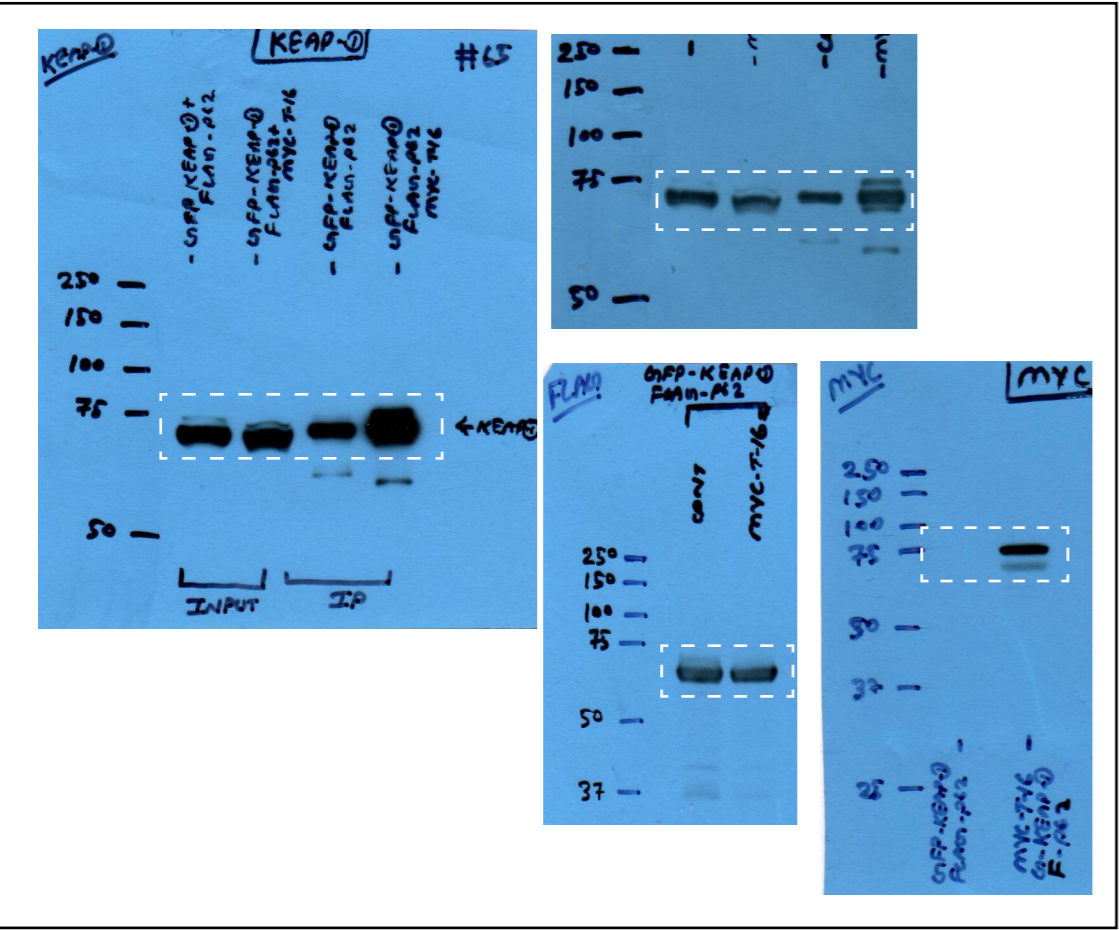

Supplement: Supplementary file 7 — Source Data for Figure 3 [file EMBJ-37-e98358-s005.pdf]

Figure 4

Panel A

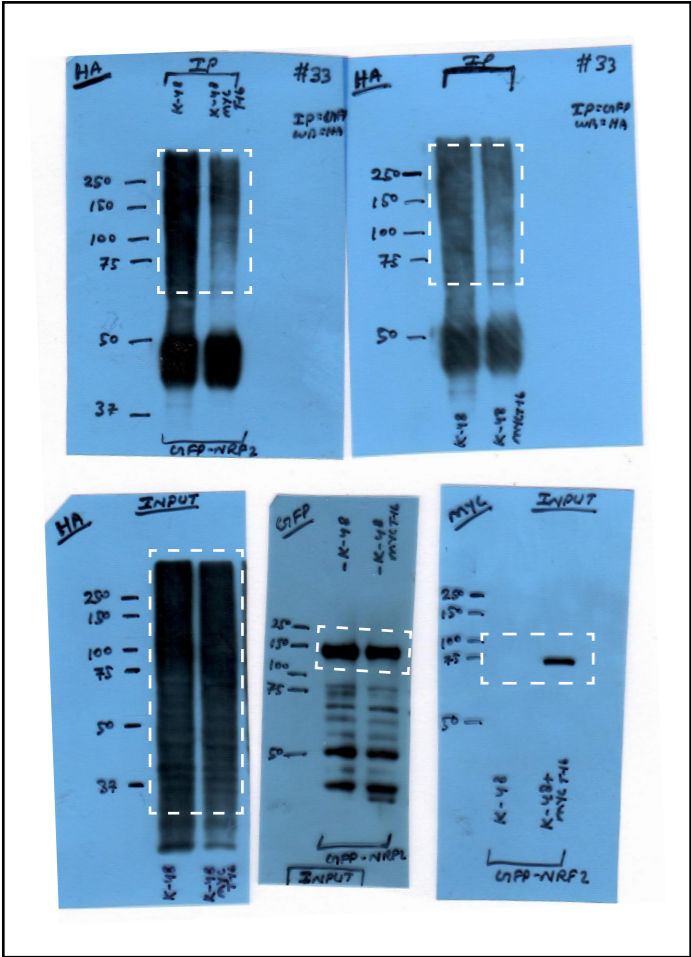

Panel B

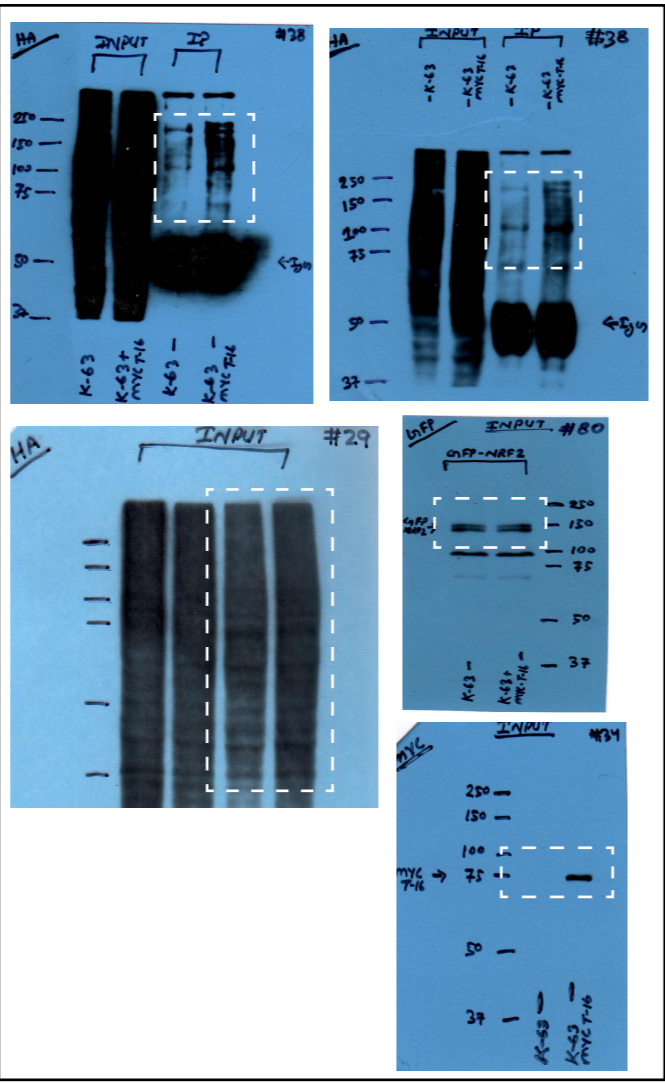

Panel C

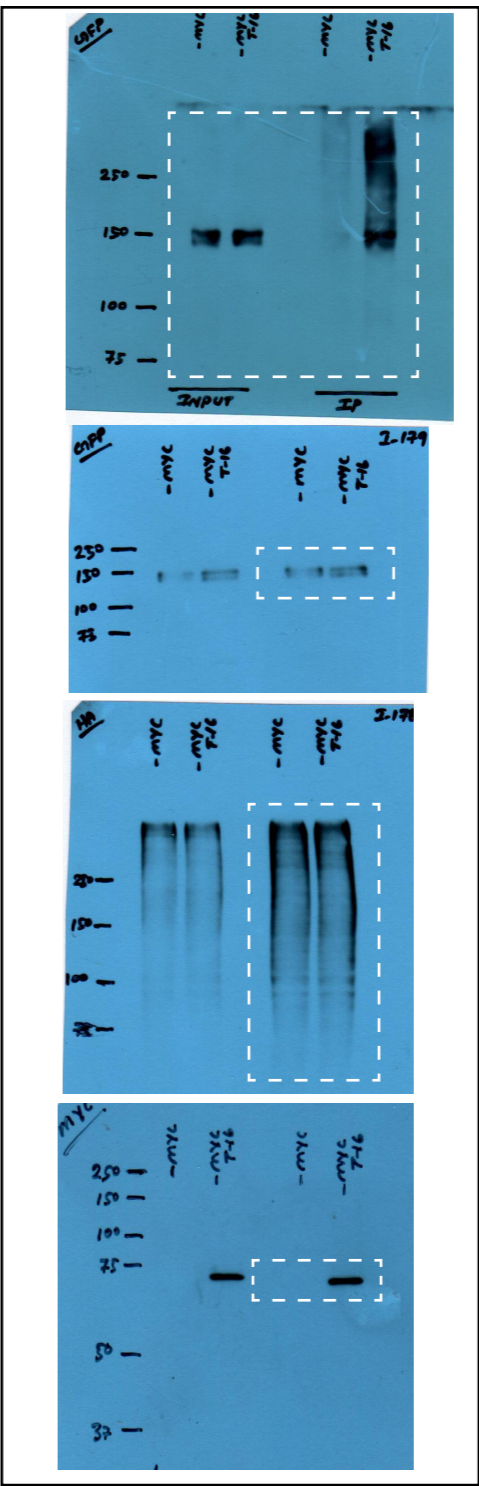

Panel D

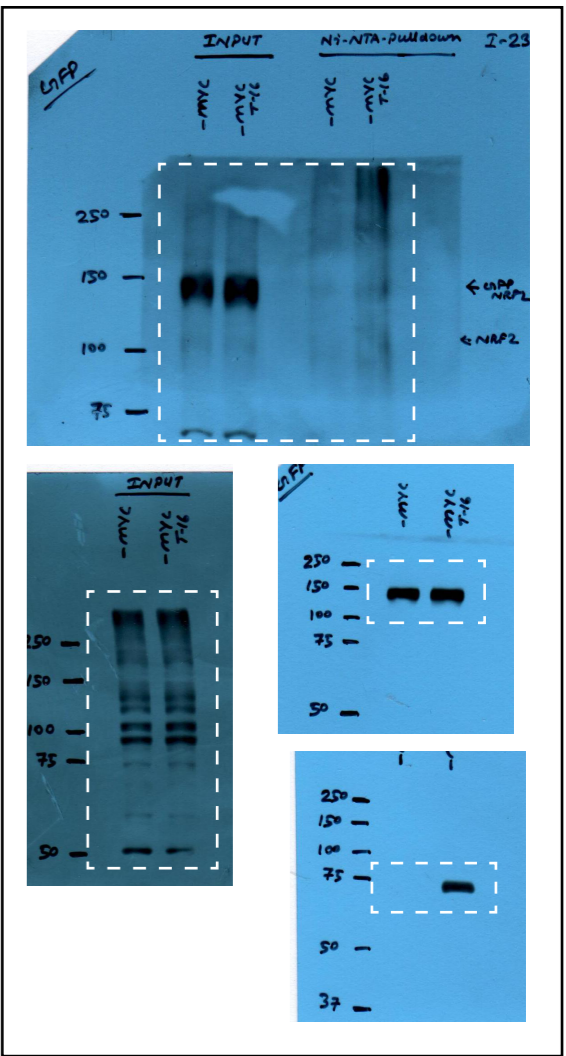

Panel E

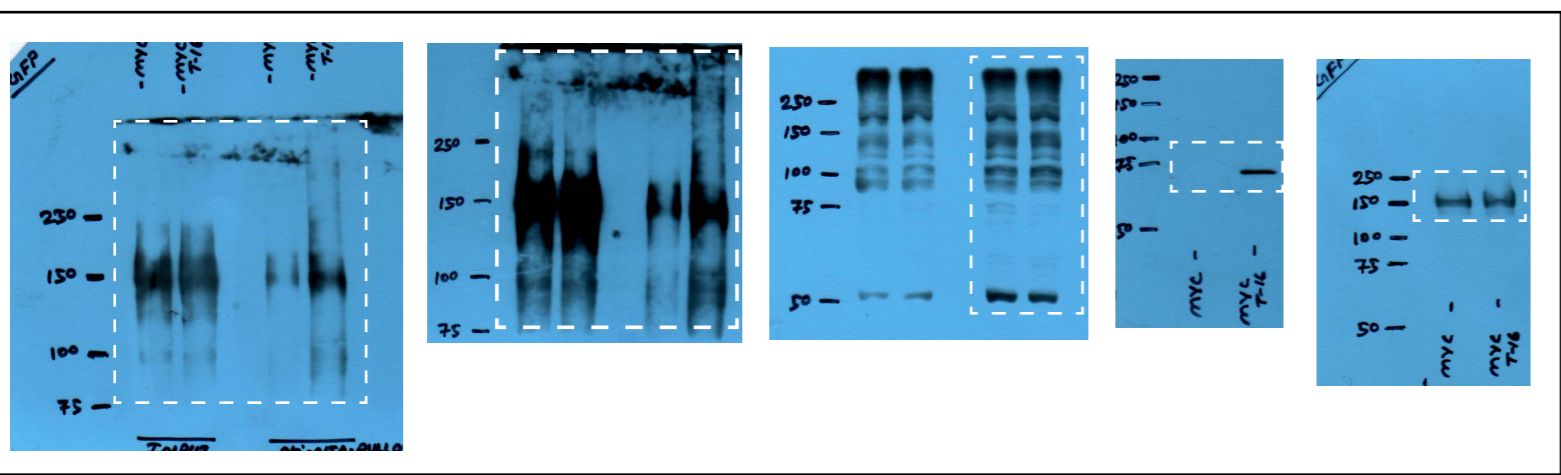

Panel F

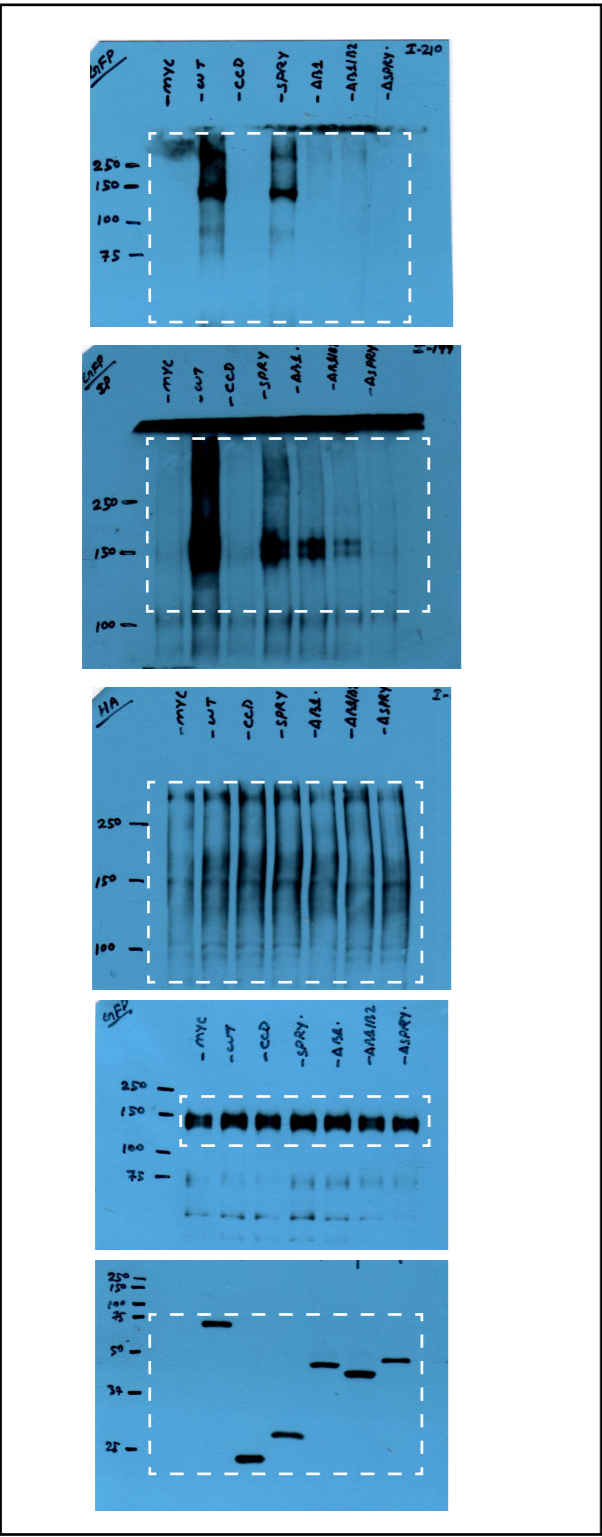

Panel G

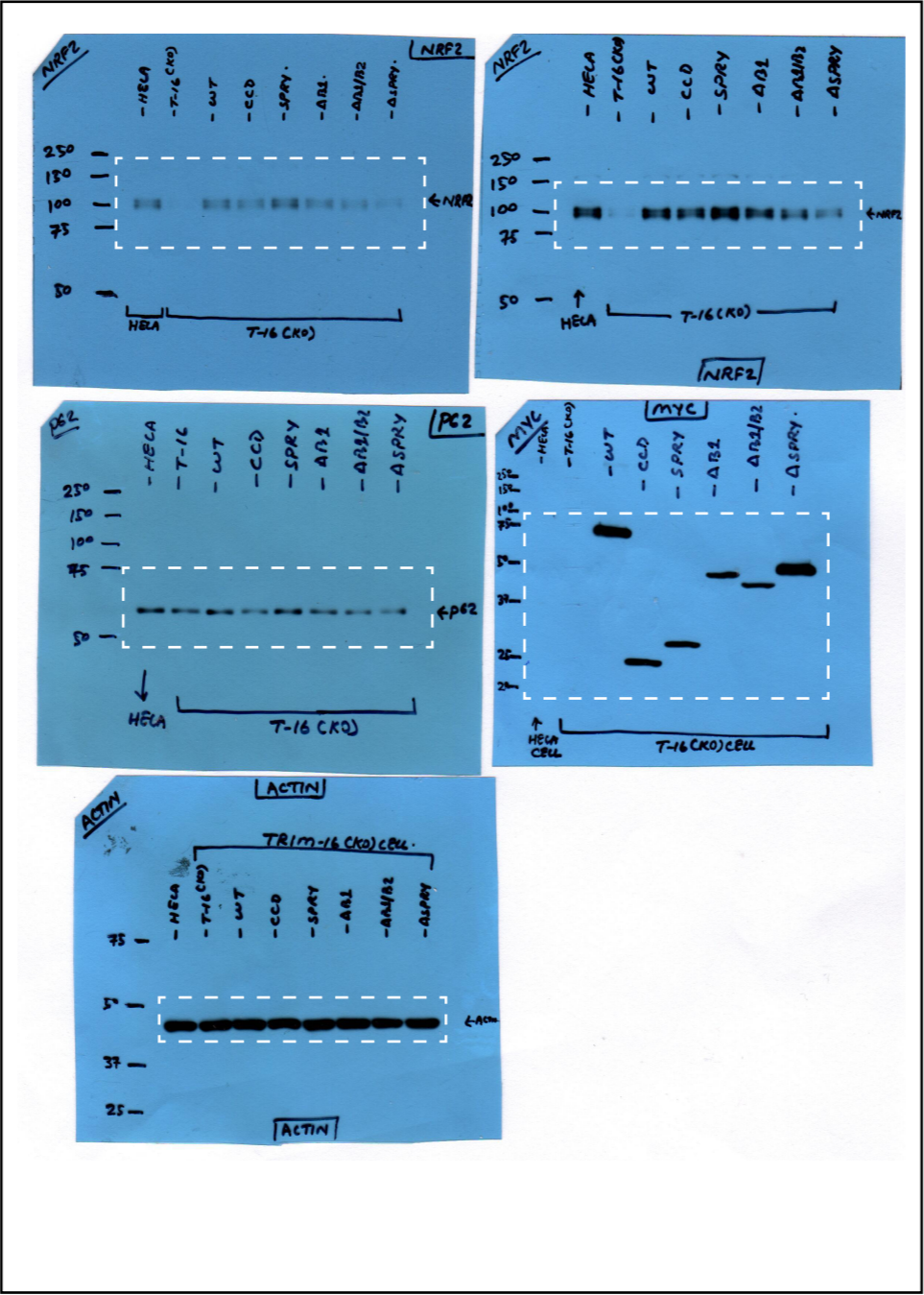

Panel H

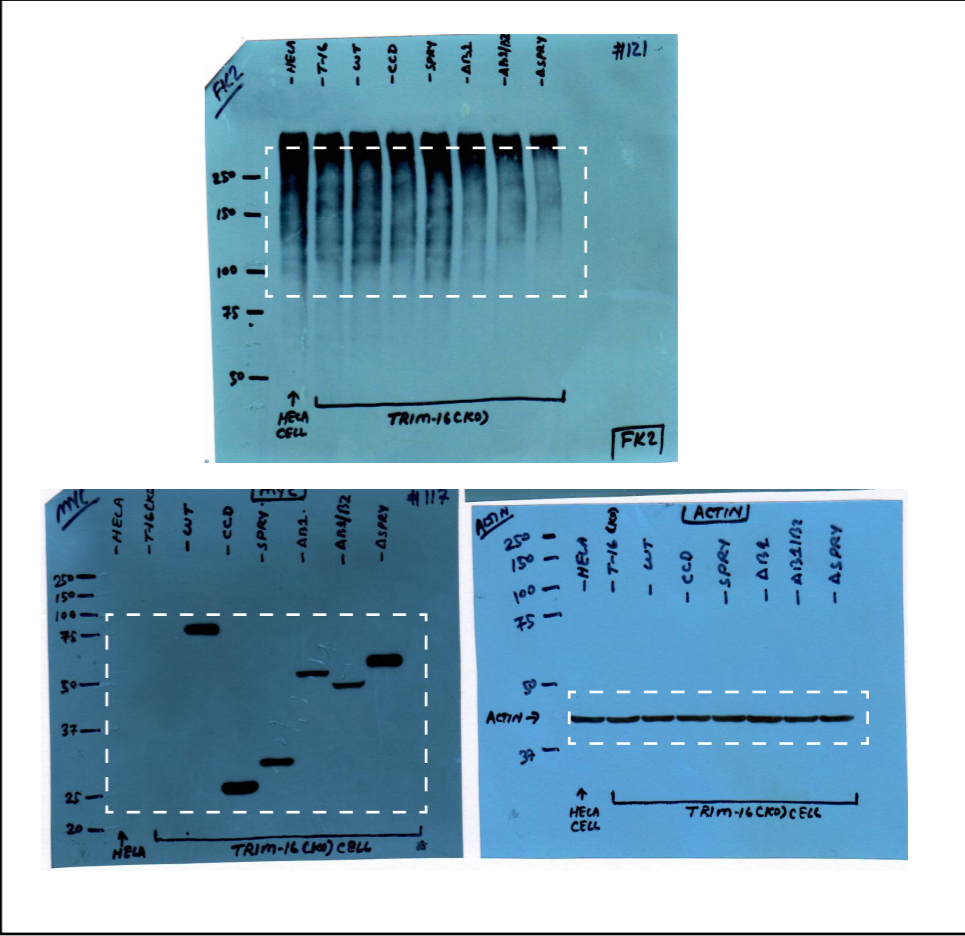

Supplement: Supplementary file 8 — Source Data for Figure 4 [file EMBJ-37-e98358-s006.pdf]

Figure 5

Panel A

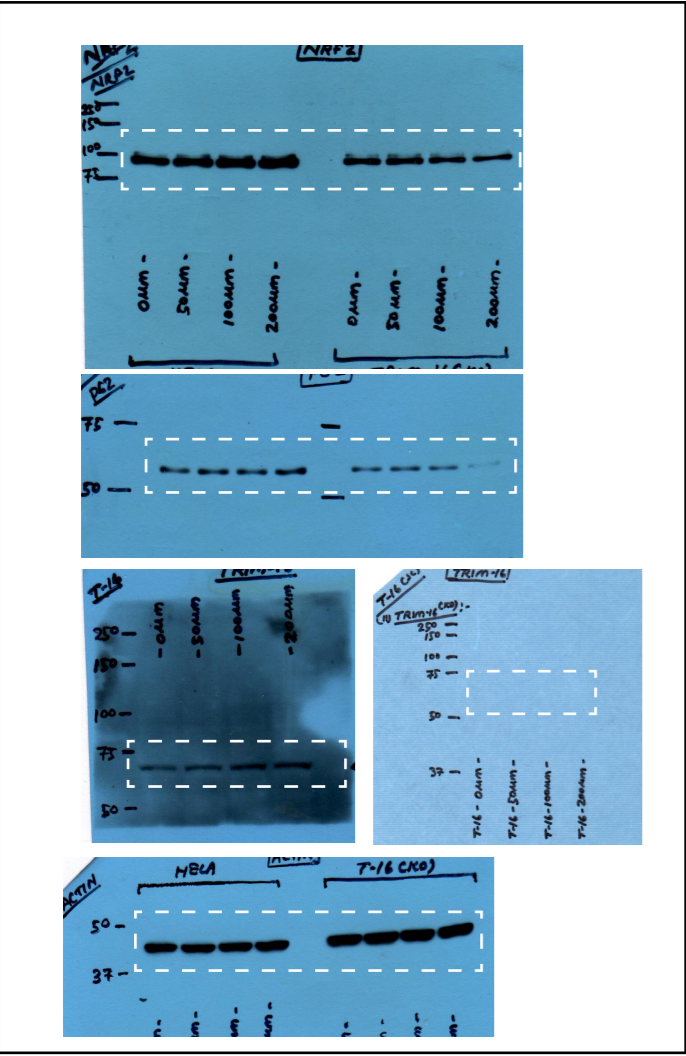

Panel B

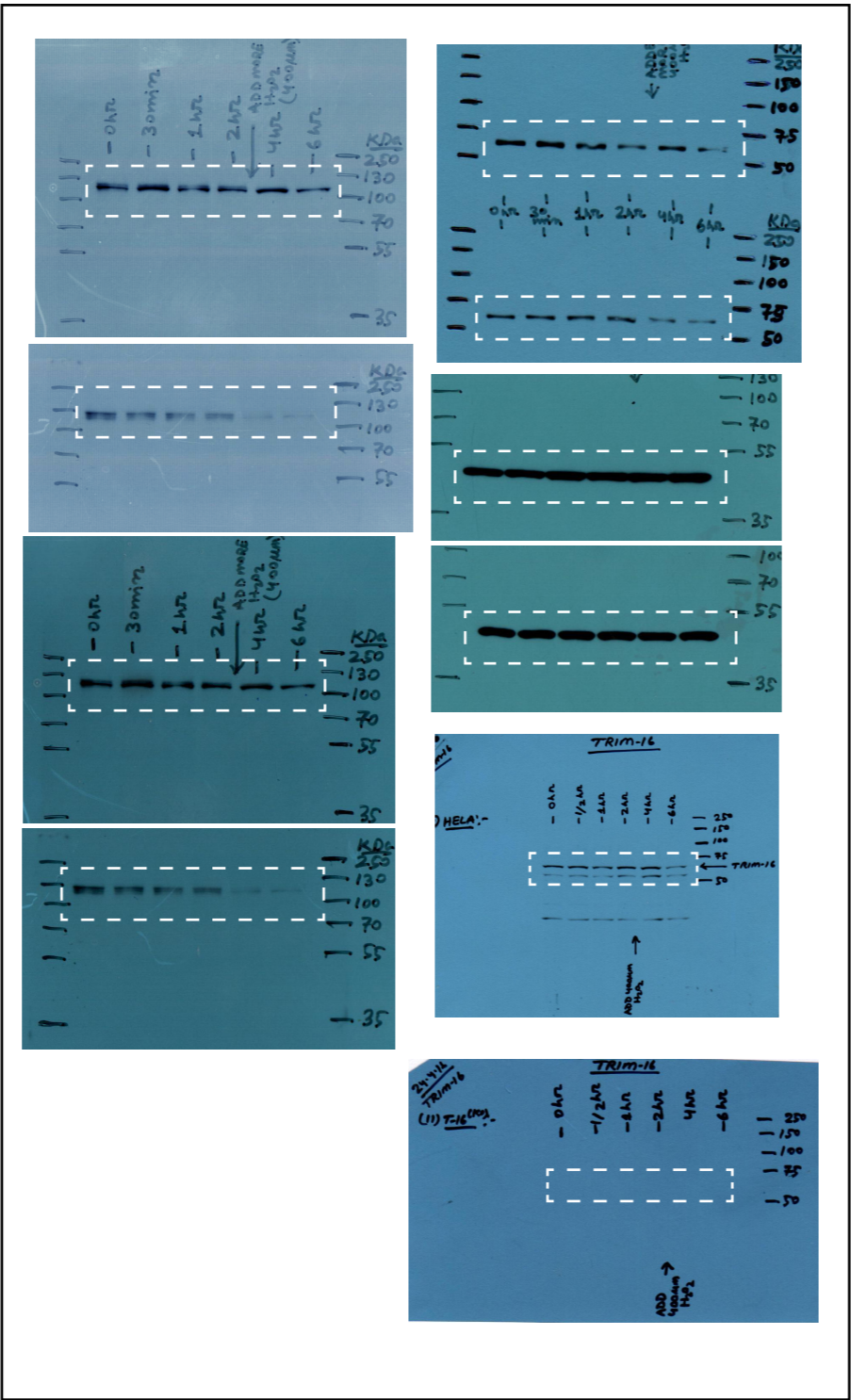

Panel D

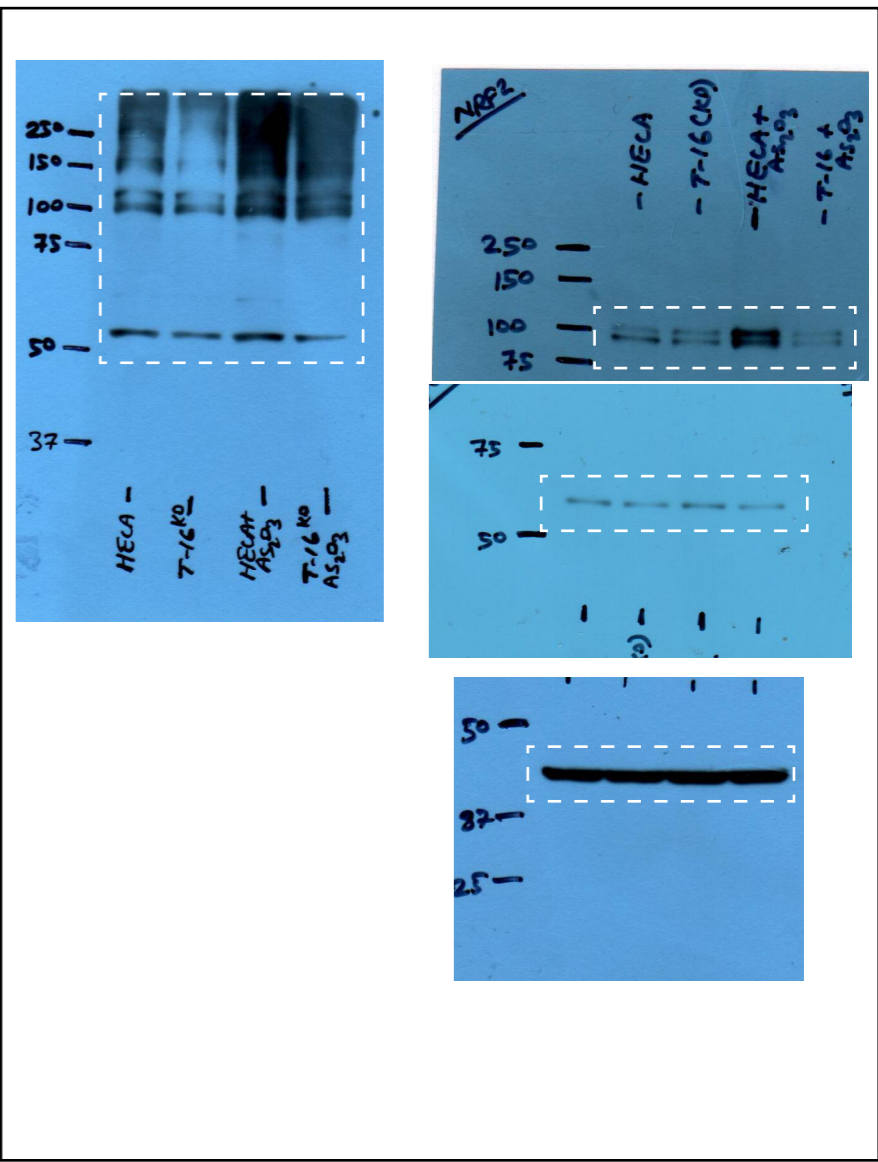

Panel K

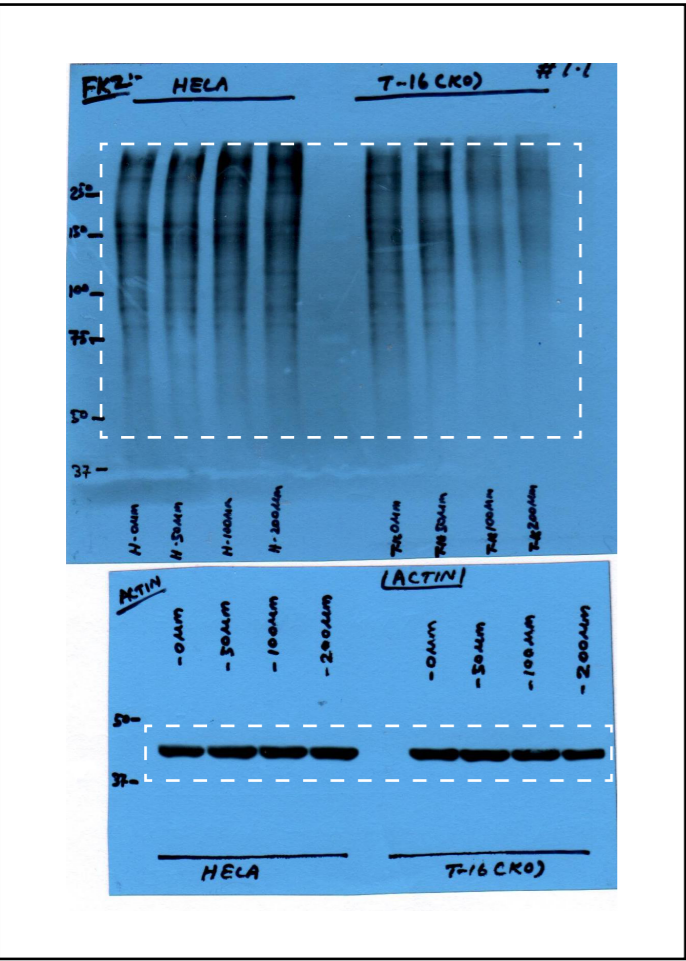

Panel O

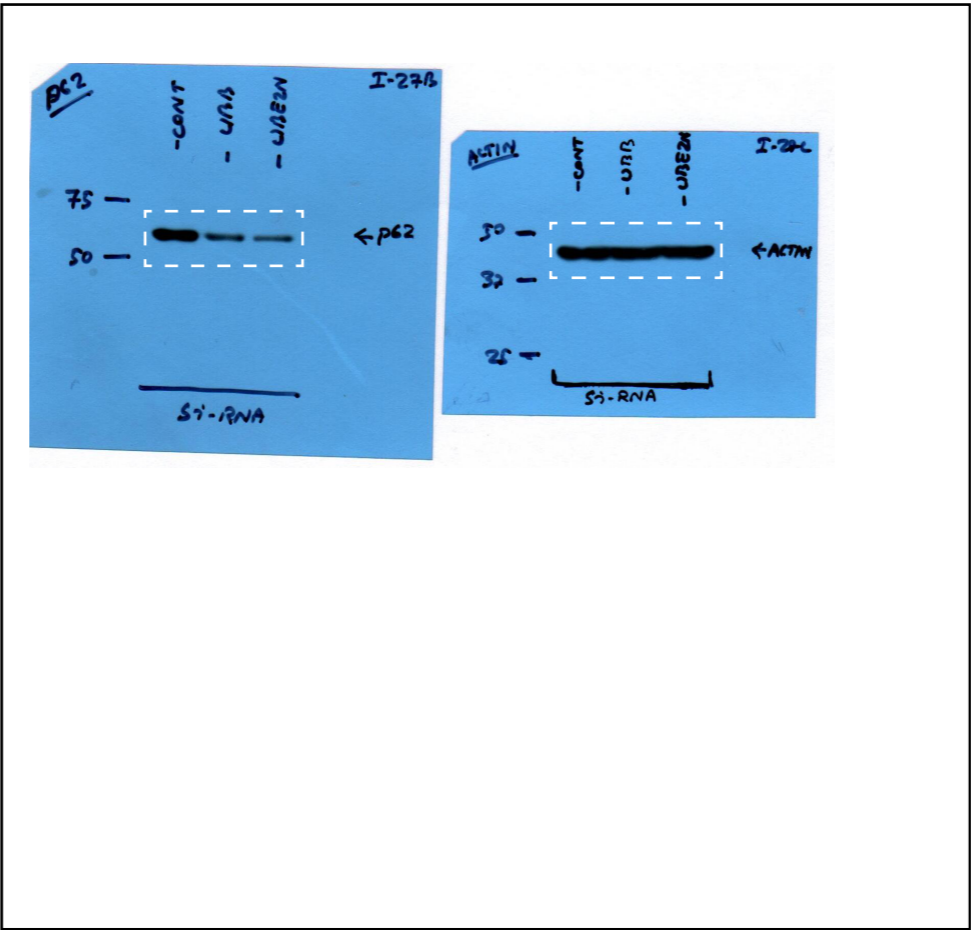

Supplement: Supplementary file 9 — Source Data for Figure 5 [file EMBJ-37-e98358-s007.pdf]

Figure 6

Panel A

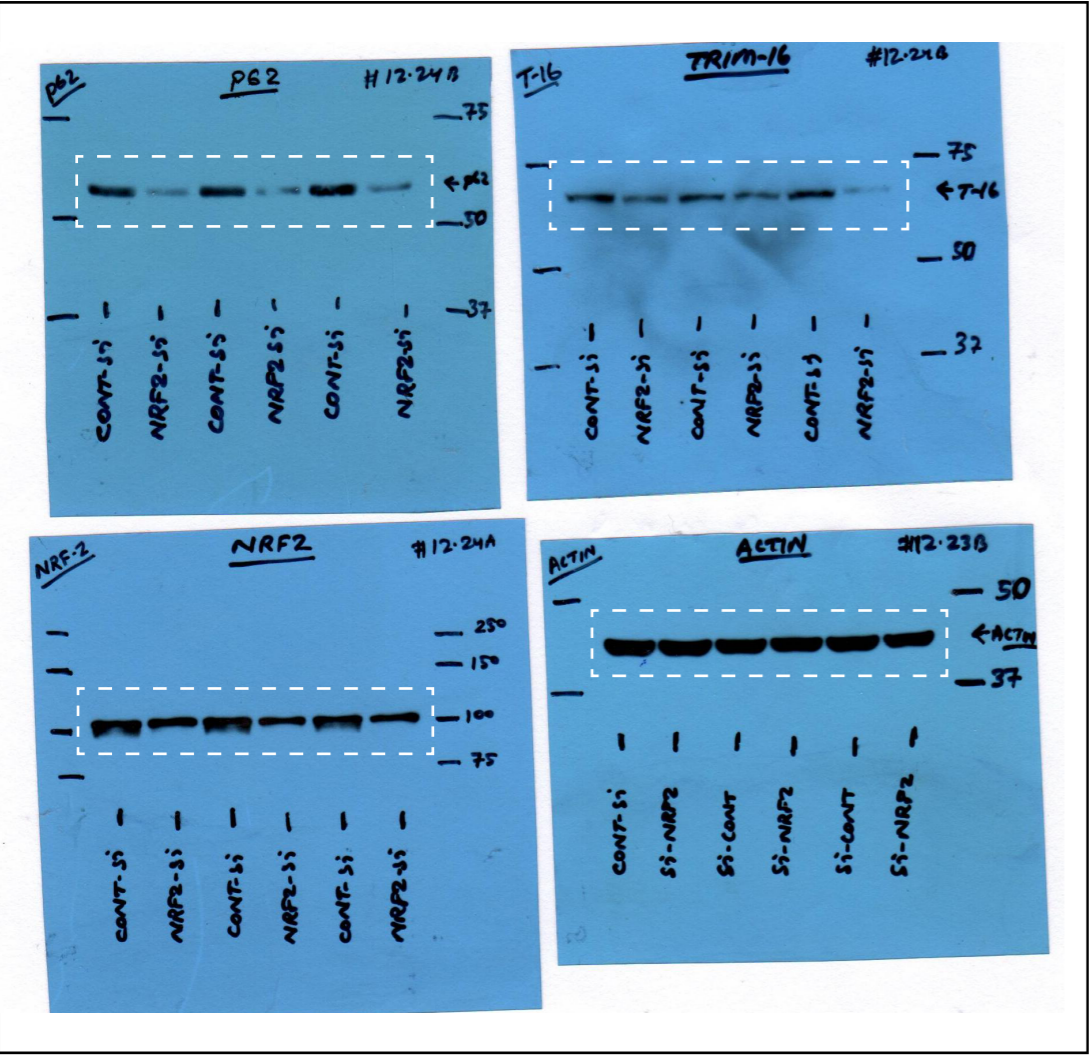

Panel D

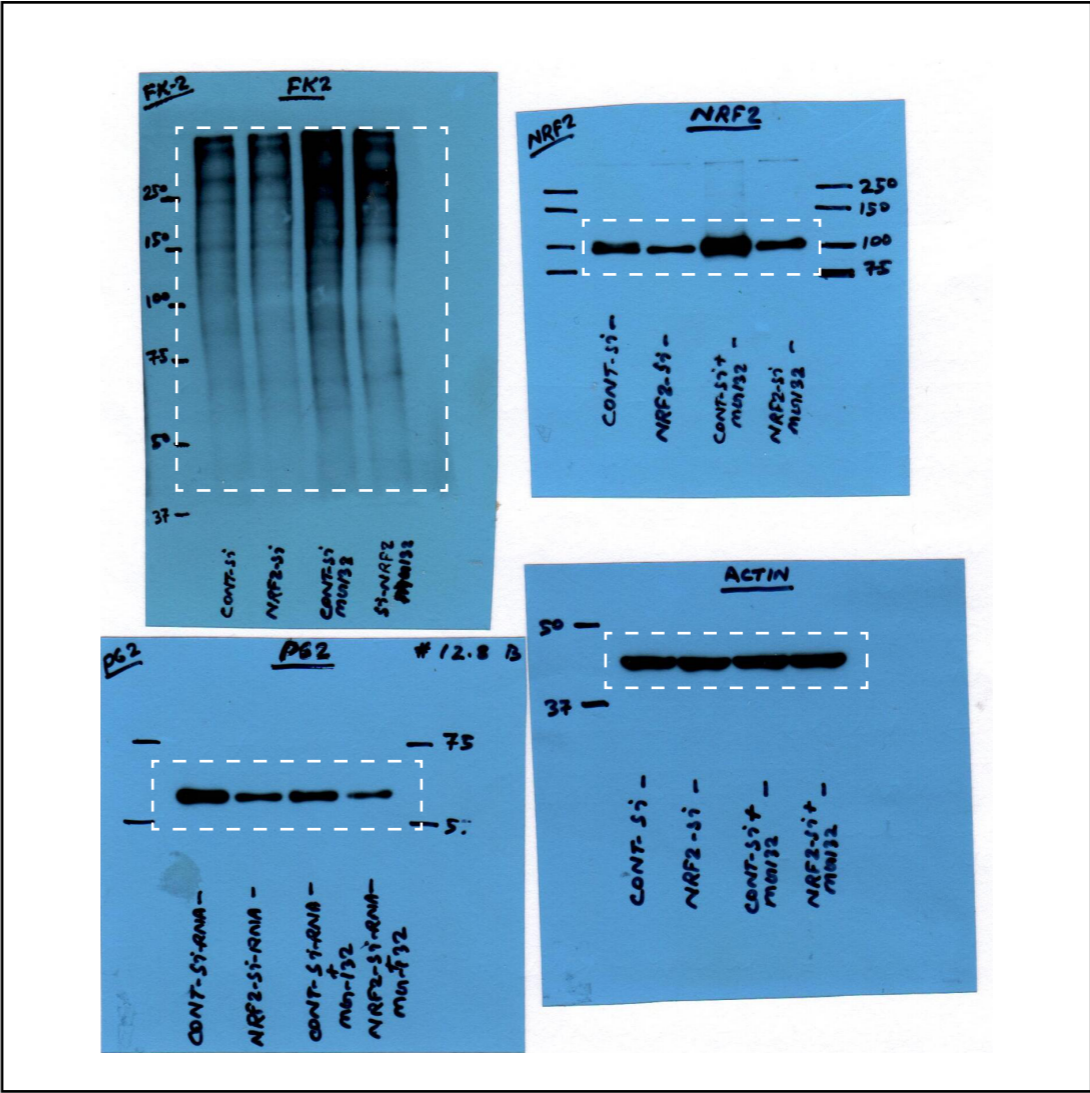

Panel E

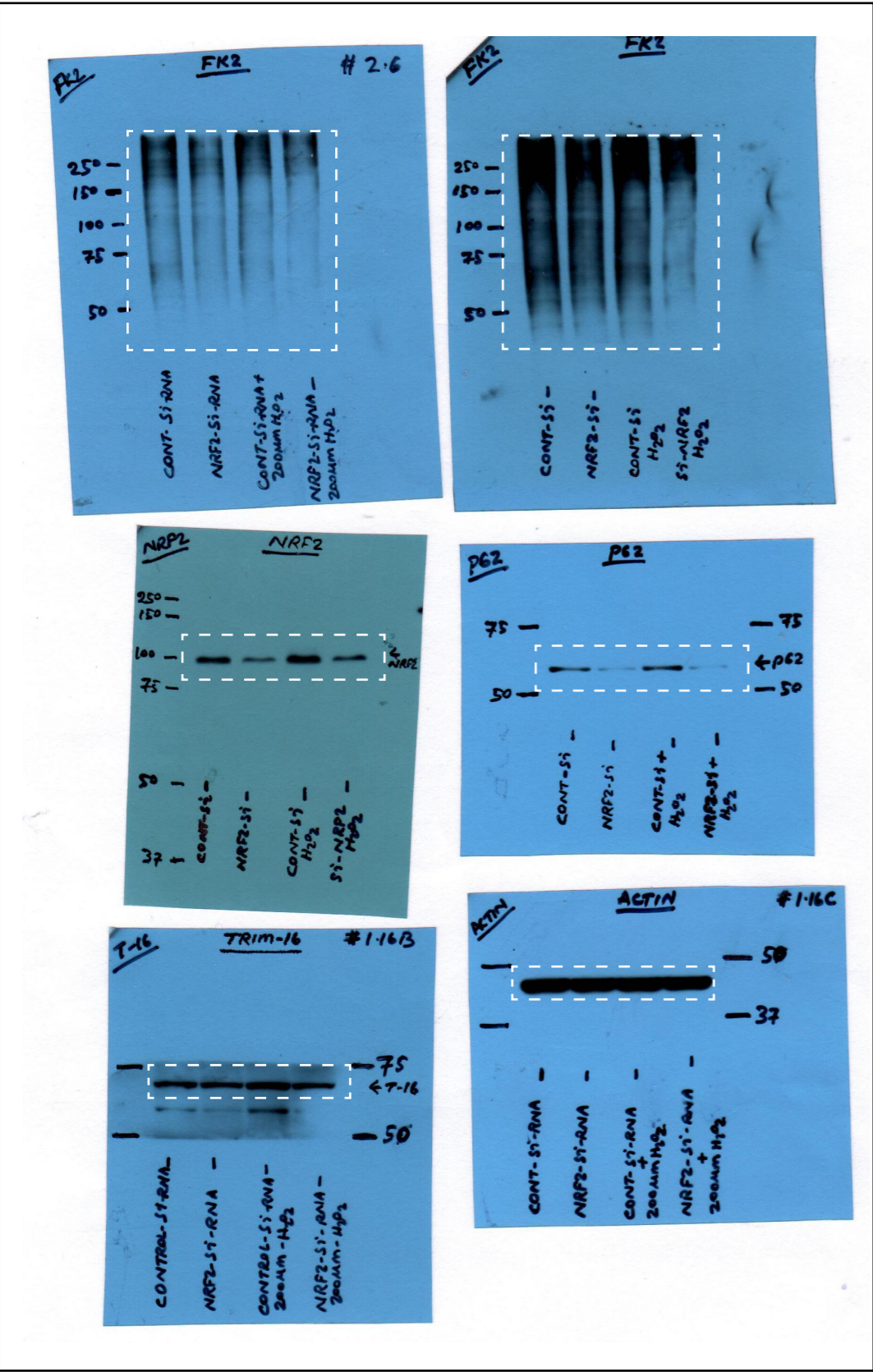

Panel H

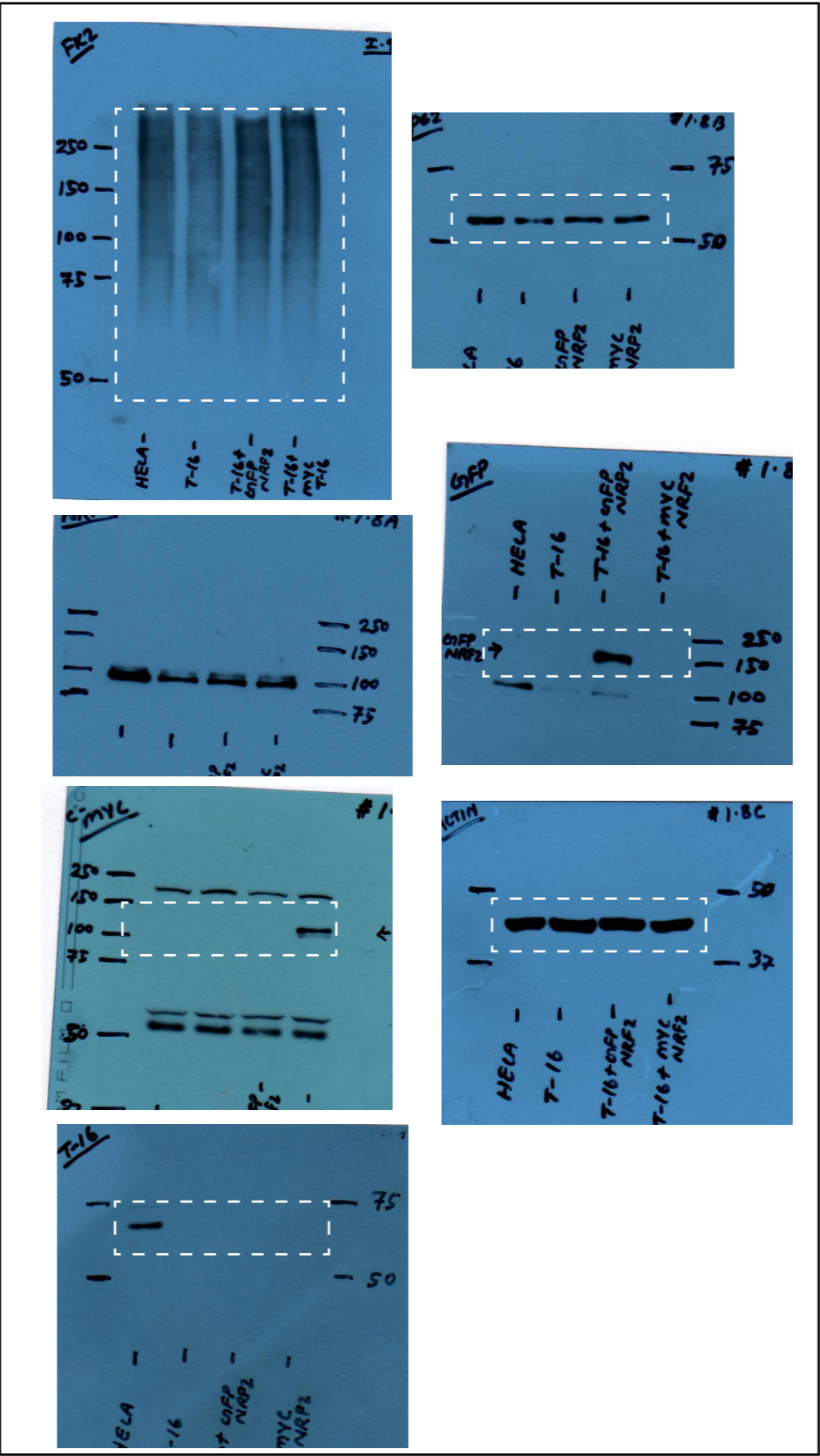

Supplement: Supplementary file 10 — Source Data for Figure 6 [file EMBJ-37-e98358-s008.pdf]

Figure 7

Panel A

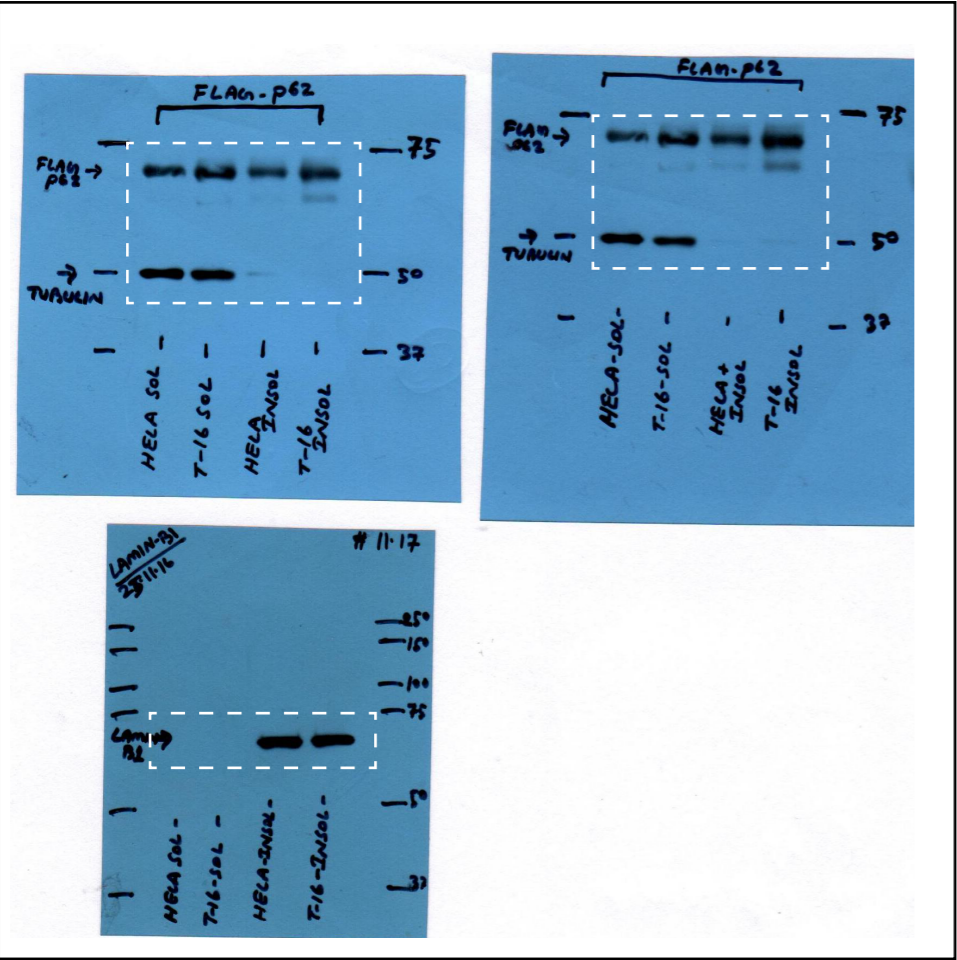

Panel C

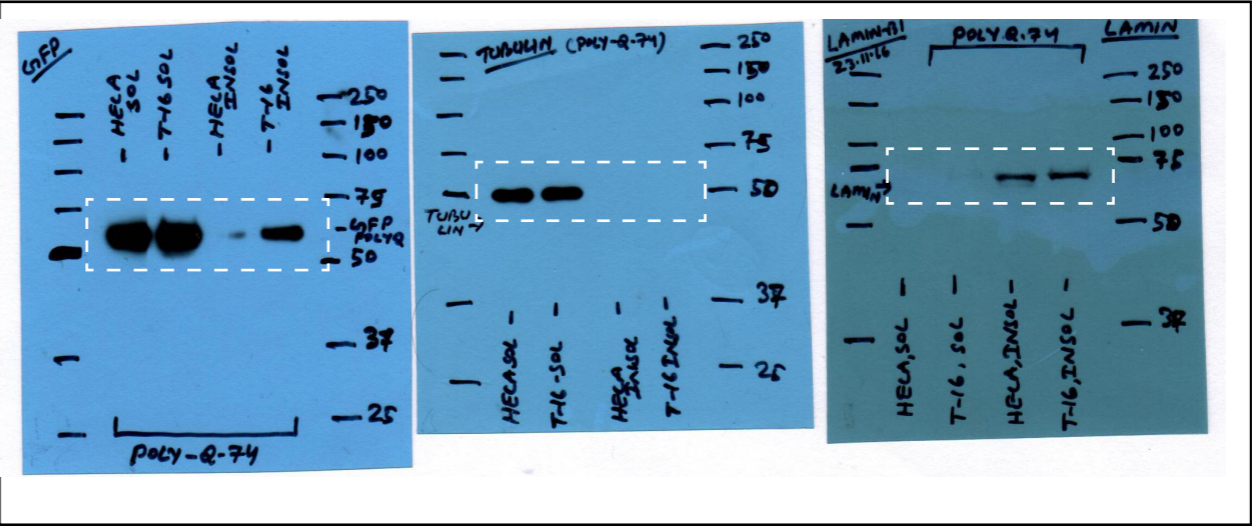

Panel H

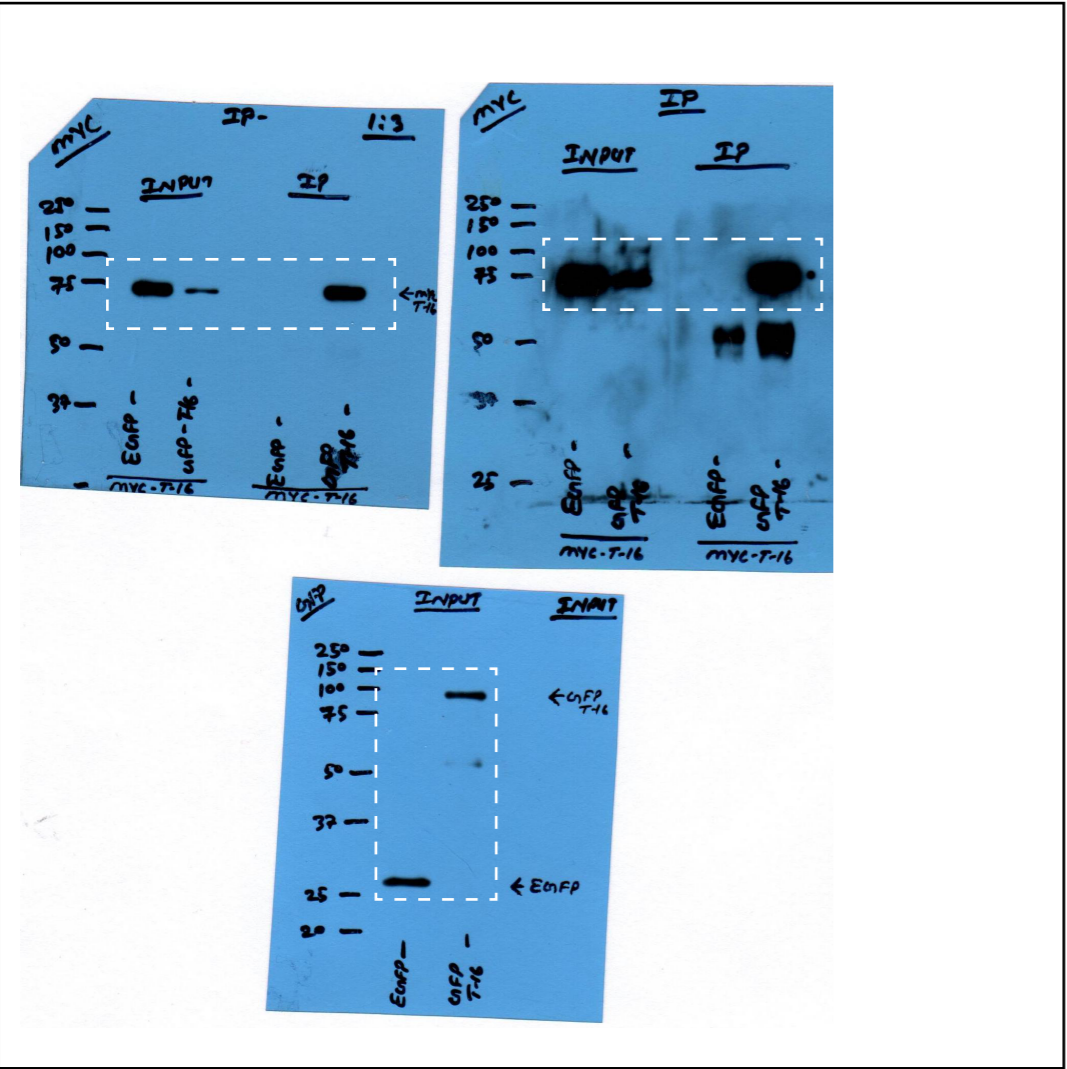

Panel I

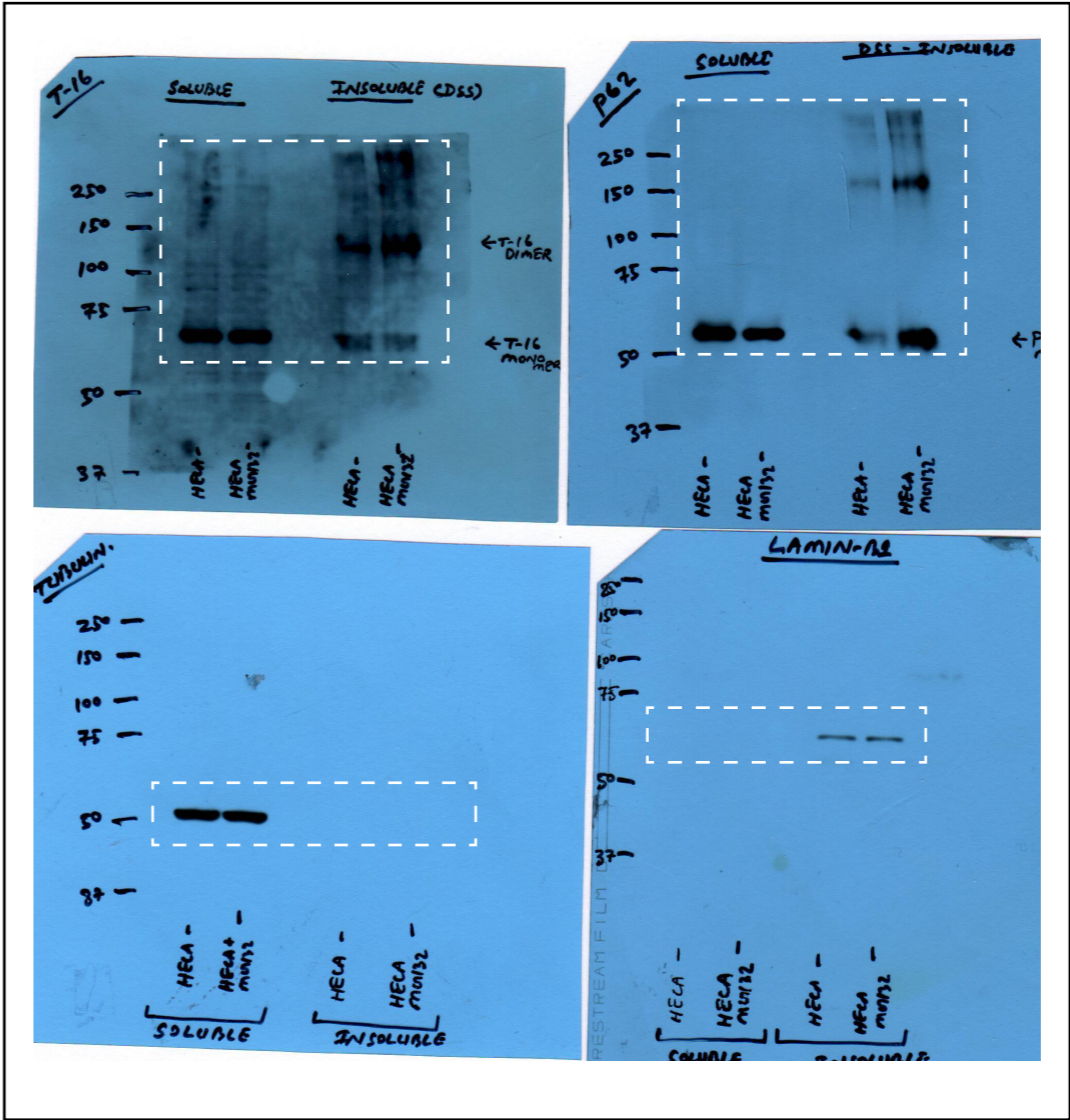

Panel J

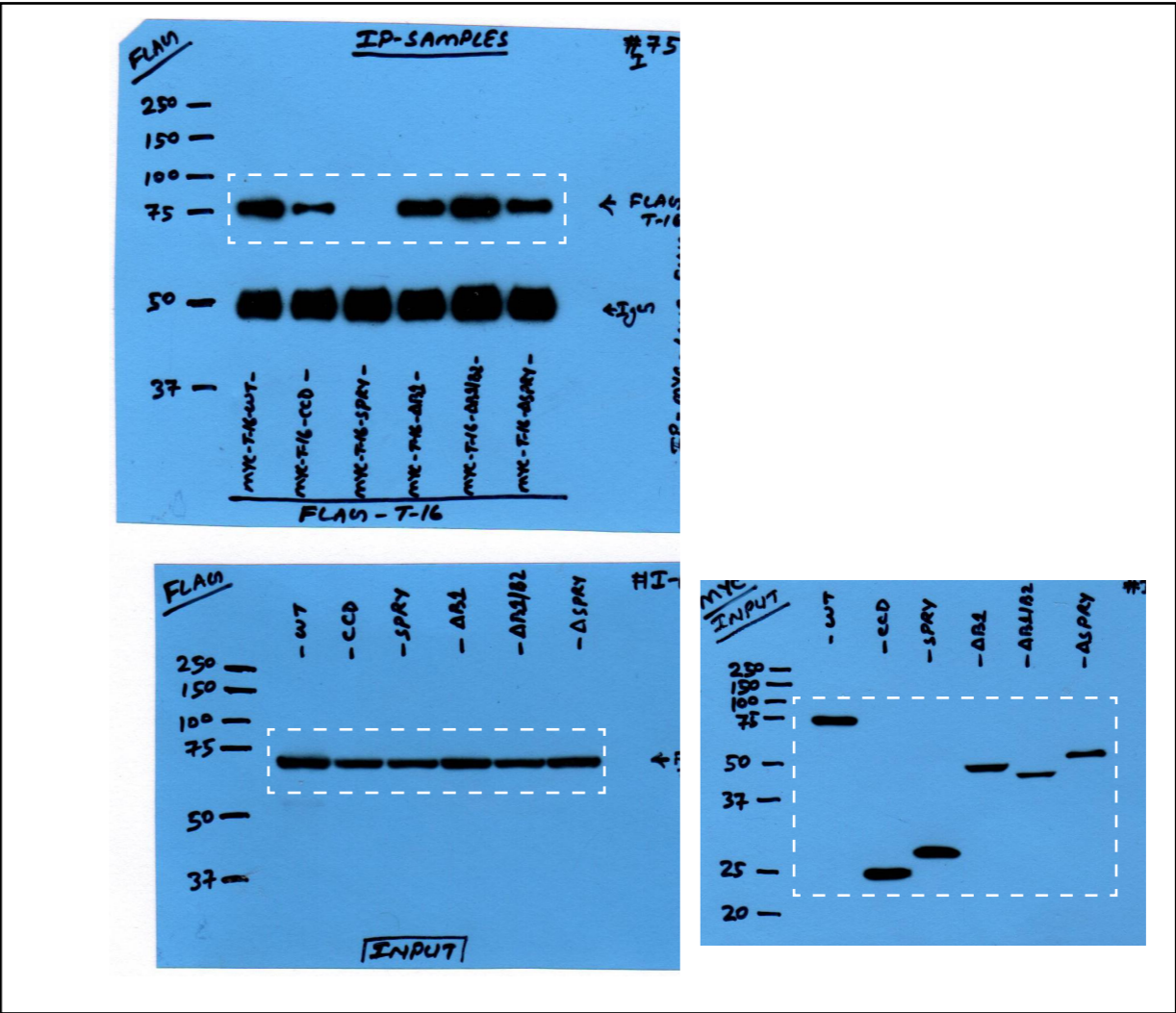

Supplement: Supplementary file 11 — Source Data for Figure 7 [file EMBJ-37-e98358-s009.pdf]

### Panel G

### Panel G

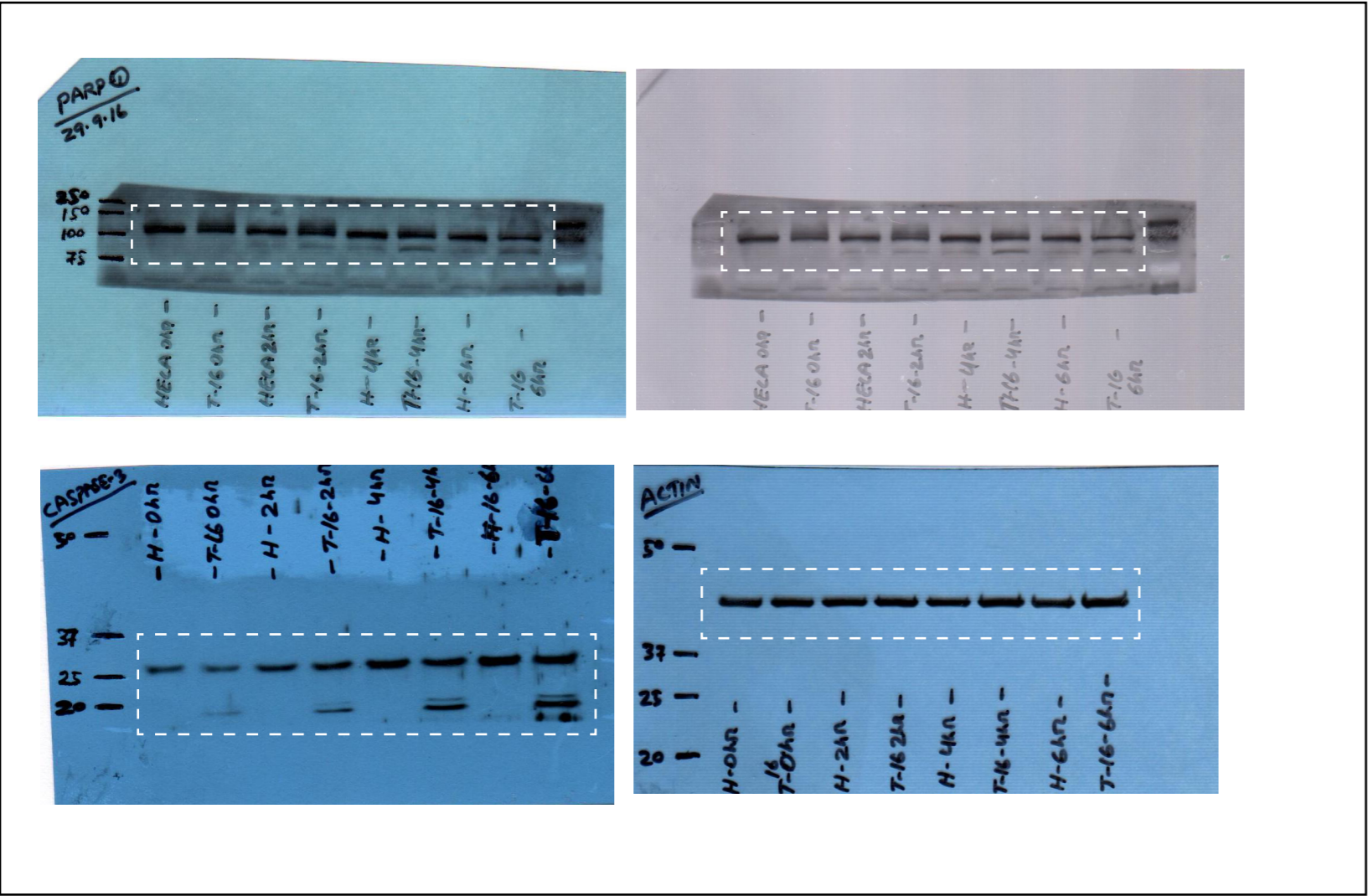

Supplement: Supplementary file 12 — Source Data for Figure 8 [file EMBJ-37-e98358-s010.pdf]

### Panel G

ELCA PA

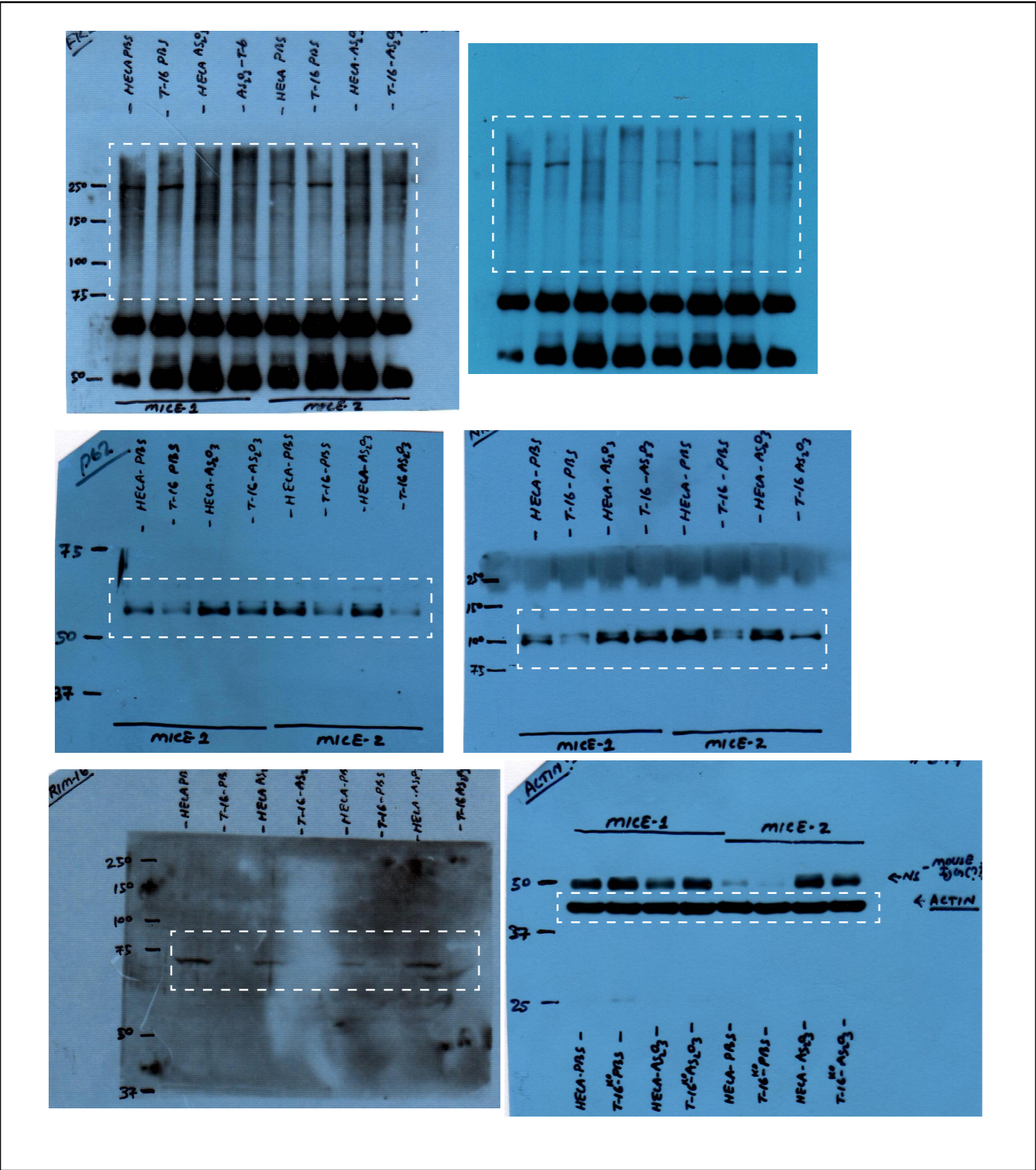

Supplement: Supplementary file 13 — Source Data for Figure 9 [file EMBJ-37-e98358-s011.pdf]
